# Low Skeletal Muscle Index Predicts Neoadjuvant Immunochemotherapy Tolerance and Survival in Oesophageal Squamous Cell Carcinoma

**DOI:** 10.1002/jcsm.70392

**Published:** 2026-09-30

**Authors:** Yizhou Huang, Maohui Chen, Zhenyuan Yang, Bingqiang Cai, Yongcong Zhang, Hongjin Wang, Shuliang Zhang, Taidui Zeng, Chun Chen, Bin Zheng

**Affiliations:** ^1^ Department of Thoracic Surgery Fujian Medical University Union Hospital Fuzhou China; ^2^ Key Laboratory of Cardio‐Thoracic Surgery (Fujian Medical University) Fujian Province University Fuzhou China; ^3^ National Key Clinical Specialty of Thoracic Surgery Fuzhou China; ^4^ Clinical Research Center for Thoracic Tumors of Fujian Province Fuzhou China; ^5^ Department of Thoracic Surgery The First Affiliated Hospital of Xiamen University Fujian China; ^6^ Department of Thoracic Surgery Quanzhou First Hospital Fujian China; ^7^ Department of Thoracic Surgery Longyan First Hospital Fujian China

**Keywords:** low skeletal muscle mass, neoadjuvant immunochemotherapy, oesophageal squamous cell carcinoma, prognosis, skeletal muscle index, treatment tolerance

## Abstract

**Background:**

Low skeletal muscle index (SMI) may identify host vulnerability in patients receiving intensive cancer therapy. However, its association with treatment tolerance and outcomes during neoadjuvant immunochemotherapy (nICT) for oesophageal squamous cell carcinoma (OSCC) remains unclear.

**Methods:**

This multicentre retrospective cohort included 461 patients with locally advanced OSCC who initiated nICT between 2020 and 2023; 404 underwent oesophagectomy. Pretreatment SMI at the third lumbar vertebra was measured by two blinded radiologists. Low SMI was defined using sex‐specific outcome‐derived cutoffs, with sensitivity analyses using published thresholds. Neoadjuvant treatment tolerance grade (NAT‐TG) was used as a composite measure of treatment tolerance and feasibility, integrating treatment modification and failure to undergo curative surgery.

**Results:**

Among 461 patients (mean age, 61.2 ± 7.3 years; 21.3% female), low SMI was present in 121 (26.2%) and was independently associated with an unfavourable NAT‐TG composite (NAT‐TG2–3 vs. NAT‐TG1; adjusted OR, 2.48; 95% CI, 1.62–3.82; *p* < 0.001). The association persisted after excluding six progression‐related surgical omissions (adjusted OR, 2.51; 95% CI, 1.59–3.95; *p* < 0.001). The overall distribution of treatment‐related adverse event severity differed between groups (*p* = 0.012), with grade ≥ 3 events occurring in 36.4% of patients with low SMI and 22.9% of those without low SMI. In the surgical population, low SMI was independently associated with severe postoperative complications (adjusted OR, 2.92; 95% CI, 1.51–5.63; *p* = 0.001). During a median follow‐up of 43 months, 165 deaths occurred. The 1‐year mortality rate in the intention‐to‐treat population was 8.5% (95% CI, 5.9%–11.0%). In the primary intention‐to‐treat model, low SMI was associated with poorer overall survival (adjusted HR, 2.36; 95% CI, 1.67–3.33; *p* < 0.001). Associations were also observed for surgical overall survival (adjusted HR, 1.91; 95% CI, 1.31–2.80) and disease‐free survival (adjusted HR, 1.57; 95% CI, 1.11–2.22). Results were generally consistent when published SMI thresholds were applied.

**Conclusions:**

Pretreatment low SMI was associated with less favourable nICT tolerance and feasibility, a higher incidence of severe postoperative complications and poorer survival. CT‐derived skeletal muscle assessment may complement baseline risk stratification, while intervention strategies require prospective validation.

## 1 Introduction

Oesophageal cancer remains a leading cause of cancer‐related mortality worldwide, with oesophageal squamous cell carcinoma (OSCC) being the predominant histological subtype in China [1]. Recent prospective evidence has established neoadjuvant immunochemotherapy (nICT) followed by surgery as a promising treatment option for locally advanced OSCC, significantly improving pathological response and long‐term survival [2, 3, 4]. However, treatment tolerance remains a substantial clinical hurdle. Data from the phase III ESCORT‐Neo trial revealed that even in highly selected cohorts, 10.8%–13.6% of patients fail to undergo surgical resection, with treatment‐related adverse events and clinical intolerability emerging as significant, non‐negligible contributors to this attrition [2]. These findings were mirrored in the REVO study, where 11.5% of patients failed to complete planned neoadjuvant cycles [5], while real‐world evidence indicates even higher rates of severe toxicity (approximately 25%–30%) and an increased inability to proceed to timely surgery [4, 6]. Notably, in most studies, analyses were restricted to patients who ultimately underwent resection, thereby excluding early intolerance and introducing selection bias. As an early and potentially modifiable determinant of treatment feasibility, tolerance represents a critical yet underexplored endpoint in the management of locally advanced OSCC.

Beyond tumour‐related factors, host nutritional and functional status has emerged as a critical determinant of treatment feasibility and oncologic outcomes [7]. Although body mass index (BMI) is widely used in routine practice, it cannot be used to differentiate between adipose tissue and lean body mass, limiting its value in reflecting true metabolic and functional reserve [8, 9]. Accumulating evidence indicates that substantial skeletal muscle depletion may be present even among patients with normal or elevated BMI [10]. These individuals are particularly susceptible to treatment‐related toxicities, dose‐limiting adverse events and premature treatment discontinuation when exposed to intensive systemic therapies, ultimately leading to inferior disease‐free and overall survival (OS) [11, 12].

In this context, imaging‐based body composition assessment has gained increasing attention in precision oncology [13]. Computed tomography, which is routinely performed for staging and surveillance in OSCC, offers an objective and reproducible approach for evaluating body composition. Among available parameters, the skeletal muscle index (SMI) measured at the third lumbar vertebra (L3) level is widely accepted as a reliable surrogate for whole‐body skeletal muscle mass [14, 15]. Previous studies have consistently demonstrated that low skeletal muscle mass is associated with increased postoperative morbidity and poorer long‐term survival in patients with oesophageal cancer [16, 17]. Furthermore, preserved skeletal muscle mass has been proposed as an important determinant underlying the so‐called obesity paradox, whereby survival advantages observed in patients with higher BMI may primarily reflect preserved skeletal muscle rather than excess adiposity per se [12].

This multicentre real‐world study aimed to evaluate the associations of pretreatment CT‐derived SMI with nICT tolerance, postoperative complications and survival in patients with locally advanced OSCC. We also explored the association between postoperative adjuvant immunotherapy and survival according to pretreatment low‐SMI status. By including both the intention‐to‐treat (ITT) and surgical populations, this study sought to characterize the clinical relevance of skeletal muscle depletion across the neoadjuvant‐to‐surgery pathway.

## 2 Methods

### 2.1 Study Design and Patient Population

This multicentre retrospective cohort study consecutively included patients with oesophageal squamous cell carcinoma treated between January 2020 and December 2023 at Fujian Medical University Union Hospital, Quanzhou First Hospital, The First Affiliated Hospital of Xiamen University and Longyan First Hospital. All patients received nICT and were scheduled for radical oesophagectomy. The ITT population comprised all eligible patients who initiated nICT, whereas the surgical population was restricted to patients who subsequently underwent oesophagectomy.

Eligible patients were those who met the following criteria: histologically confirmed OSCC; locally advanced disease (cT3–4aN0–3M0 or cT1–2N+M0) according to the American Joint Committee on Cancer (AJCC) 8th edition staging system; receipt of a programmed cell death protein 1 (PD‐1) inhibitor combined with platinum‐based doublet chemotherapy; and availability of a pretreatment abdominal CT scan within 2 weeks before therapy with sufficient quality for body composition analysis. The exclusion criteria included a history of other malignancies; severe active autoimmune disease or long‐term systemic corticosteroid therapy that precluded PD‐1 inhibitor treatment; inadequate CT coverage at the L3 level; and incomplete follow‐up data. Patients who achieved a clinical complete response and elected active surveillance, as well as those who declined surgery solely for personal or financial reasons unrelated to treatment toxicity or functional deterioration were excluded.

The study was conducted in accordance with the Declaration of Helsinki. The protocol was approved by the Institutional Review Board of Fujian Medical University Union Hospital (Approval No. 2025KY671), which waived the requirement for individual informed consent because of the retrospective design. Formal data‐sharing permissions and local institutional authorizations were obtained from the other participating centres before the transfer and centralized analysis of anonymized clinical and imaging data.

#### 2.1.1 Treatment Protocol

PD‐1 inhibitors, including camrelizumab, sintilimab, tislelizumab, nivolumab or pembrolizumab, were administered intravenously in combination with platinum‐based doublet chemotherapy. Each treatment cycle was administered every 3 weeks. The planned nICT course comprised two or three cycles according to institutional protocols. Dose reduction, treatment delay and early discontinuation were recorded, together with PD‐1 inhibitor type, chemotherapy backbone, planned and actual cycles and treatment year, to evaluate treatment heterogeneity. Radiologic reassessment was routinely performed 4–6 weeks after the completion of neoadjuvant therapy. In the absence of contraindications, patients subsequently underwent minimally invasive McKeown or Ivor Lewis oesophagectomy based on tumour location and institutional practice, with systematic two‐field or three‐field lymphadenectomy.

### 2.2 CT‐Based Body Composition Analysis

Baseline abdominal computed tomography images were retrieved in Digital Imaging and Communications in Medicine format and analysed using dedicated medical imaging software (SliceOmatic version 5.0, Tomovision, Canada). Portal venous‐phase images were preferentially used when available. Two trained radiologists independently selected one axial image at the mid‐pedicle level of L3, where both transverse processes were clearly visible [18, 19]. Skeletal muscle was segmented using attenuation thresholds of −29 to +150 Hounsfield units, with manual boundary correction when required to exclude misclassified adjacent structures. Skeletal muscle area (SMA) was normalized to height squared to calculate SMI (cm^2^/m^2^). Both readers were blinded to clinical information, treatment course, survival outcomes and the final low‐SMI classification. The final value used for analysis was based on the mean of the two independent measurements, with significant discrepancies resolved by consensus. Interobserver agreement of the initial measurements was assessed using a two‐way mixed‐effects, absolute‐agreement intraclass correlation coefficient (ICC).

### 2.3 Definition of Low SMI

Given ethnic differences in body composition and pronounced sex‐related variability in skeletal muscle mass, fixed cutoff values derived from Western populations were not adopted. Sex‐specific optimal SMI cutoff values were determined using X‐tile software (version 3.6.1, Yale University), with OS as the endpoint. X‐tile evaluated all candidate splits and selected the cutoff yielding the maximum log‐rank chi‐square statistic. Low SMI was defined as an SMI < 51.5 cm^2^/m^2^ in men and < 37.5 cm^2^/m^2^ in women. Because muscle strength and physical performance were not assessed, low SMI is used throughout this study to describe reduced CT‐derived skeletal muscle quantity rather than a clinical diagnosis of sarcopenia. To assess the robustness of these outcome‐derived cutoffs, sensitivity analyses were repeated using the published Prado thresholds [20] (< 52.4 cm^2^/m^2^ in men and < 38.5 cm^2^/m^2^ in women) and Fearon thresholds [18] (< 55.0 cm^2^/m^2^ in men and < 39.0 cm^2^/m^2^ in women).

### 2.4 Assessment and Grading of Neoadjuvant Therapy Tolerance

To characterize treatment tolerance and feasibility across the neoadjuvant‐to‐surgery pathway, a neoadjuvant treatment tolerance grade (NAT‐TG) was defined according to treatment intensity, treatment delay, early discontinuation and completion of curative surgery. NAT‐TG1 indicated completion of planned full‐intensity nICT followed by surgery. NAT‐TG2 indicated dose reduction, treatment delay or early discontinuation, with subsequent completion of surgery. NAT‐TG3 indicated unplanned failure to undergo curative surgery after initiation of nICT. For NAT‐TG3 patients, the dominant reason for surgical omission was classified as severe treatment‐related toxicity, persistent clinical or functional deterioration without disease progression sufficient to explain surgical omission or disease progression as the dominant reason. The primary composite endpoint was an unfavourable NAT‐TG, defined as NAT‐TG2–3 versus NAT‐TG1. A restricted sensitivity analysis excluded six NAT‐TG3 patients whose surgical omission was predominantly attributable to disease progression.

### 2.5 Exploratory Evaluation of Adjuvant Immunotherapy

The association between postoperative adjuvant immunotherapy and survival was evaluated as an exploratory analysis stratified by pretreatment low‐SMI status. Postoperative adjuvant immunotherapy was defined as treatment initiated within 3 months after surgery. In the conventional subgroup analyses, OS and disease‐free survival (DFS) were calculated from the date of surgery. Within the low‐SMI subgroup, multivariable Cox models were adjusted for age, sex, ypTNM stage and postoperative Clavien–Dindo grade. To reduce immortal time bias, a 6‐month landmark analysis included only patients who were alive and recurrence‐free at 6 months after surgery, with follow‐up recalculated from the landmark date. Treatment exposure was defined using this 3‐month postoperative window and was therefore fully ascertained before the 6‐month landmark.

### 2.6 Outcome Definitions

Treatment‐related adverse events were graded according to the Common Terminology Criteria for Adverse Events version 5.0. Postoperative complications were classified using the Clavien–Dindo system, with grade ≥III defined as severe. In the ITT population, OS was calculated from initiation of nICT to death or last follow‐up. In the surgical population, OS and DFS were calculated from surgery to death and to recurrence or death, respectively. Patients without an event were censored at the last follow‐up.

### 2.7 Statistical Analysis

All statistical analyses were performed using R version 4.4.2 (R Foundation for Statistical Computing, Vienna, Austria), with the tableone, survival, survminer, rms and tidyverse packages. Continuous variables were expressed as mean ± standard deviation or median with interquartile range and compared using Student's *t*‐test or the Mann–Whitney *U* test, as appropriate. Categorical variables were compared using the chi‐square test or Fisher's exact test. No data were missing for variables included in the principal models; therefore, no imputation was performed. Logistic regression evaluated unfavourable NAT‐TG (NAT‐TG2–3 vs. NAT‐TG1). The multivariable model included BMI, low SMI and clinical T stage and was repeated after excluding six progression‐related NAT‐TG3 cases. Severe postoperative complications were assessed using multivariable logistic regression adjusted for age, sex, BMI, serum albumin, operative approach, lymphadenectomy extent, tumour location and centre, with centre entered as a fixed effect.

Survival outcomes were estimated using the Kaplan–Meier method and compared using the log‐rank test. Two‐ and three‐year survival estimates were reported with 95% confidence intervals. Three centre‐stratified Cox models were constructed for OS in the ITT population. Model 1 included low SMI and baseline variables with univariable *p* < 0.10 (serum albumin and log_2_‐transformed systemic immune‐inflammation index). Model 2, the primary clinically specified model, additionally included age, sex, BMI, ECOG performance status and clinical TNM stage. Model 3 further included actual nICT cycles and PD‐1 inhibitor type. Pathological variables were restricted to analyses of the surgical population. Proportional hazards assumptions were evaluated using Schoenfeld residuals, and events per regression parameter were examined. Models 2 and 3 underwent internal validation using 1000 bootstrap resamples with the same covariate specifications and centre stratification. Discrimination was assessed using apparent and optimism‐corrected C‐indices, and calibration was evaluated for 3‐year OS. During bootstrap resampling, the study‐derived SMI cutoff was held fixed; bootstrap validation therefore assessed optimism‐corrected performance of the fitted Cox models. Robustness to SMI definition was evaluated separately using the published Prado and Fearon thresholds. Treatment heterogeneity was examined according to treatment year, regimen, planned cycle protocol and actual cycles received. All statistical tests were two‐sided, and *p* values < 0.05 were considered statistically significant.

## 3 Results

### 3.1 Patients' Characteristics and Identification of Low SMI

A total of 461 patients with locally advanced OSCC who received nICT were included in the analysis (Figure 1). The cohort was predominantly male (78.7%), with a mean age of 61.2 ± 7.3 years. Based on baseline computed tomography images at the third lumbar vertebra level, the mean SMA was 150.9 ± 30.9 cm^2^ and the mean SMI was 54.6 ± 9.9 cm^2^/m^2^. Using sex‐specific cutoff values derived from X‐tile analysis (< 51.5 cm^2^/m^2^ for male patients and < 37.5 cm^2^/m^2^ for female patients), 121 patients (26.2%) were classified as having low SMI (Table 1). Centre‐specific distributions of treatment year, PD‐1 inhibitor type, chemotherapy backbone and planned and actual nICT cycles are summarized in Table S1. Manual boundary refinement was required in 244 of 461 cases (52.9%) for the first radiologist and 216 of 461 cases (46.9%) for the second radiologist. Despite independent slice selection and segmentation, interobserver agreement was excellent, with an ICC of 0.994 (95% CI, 0.992–0.995) for SMA and 0.992 (95% CI, 0.990–0.993) for SMI.

**FIGURE 1:**
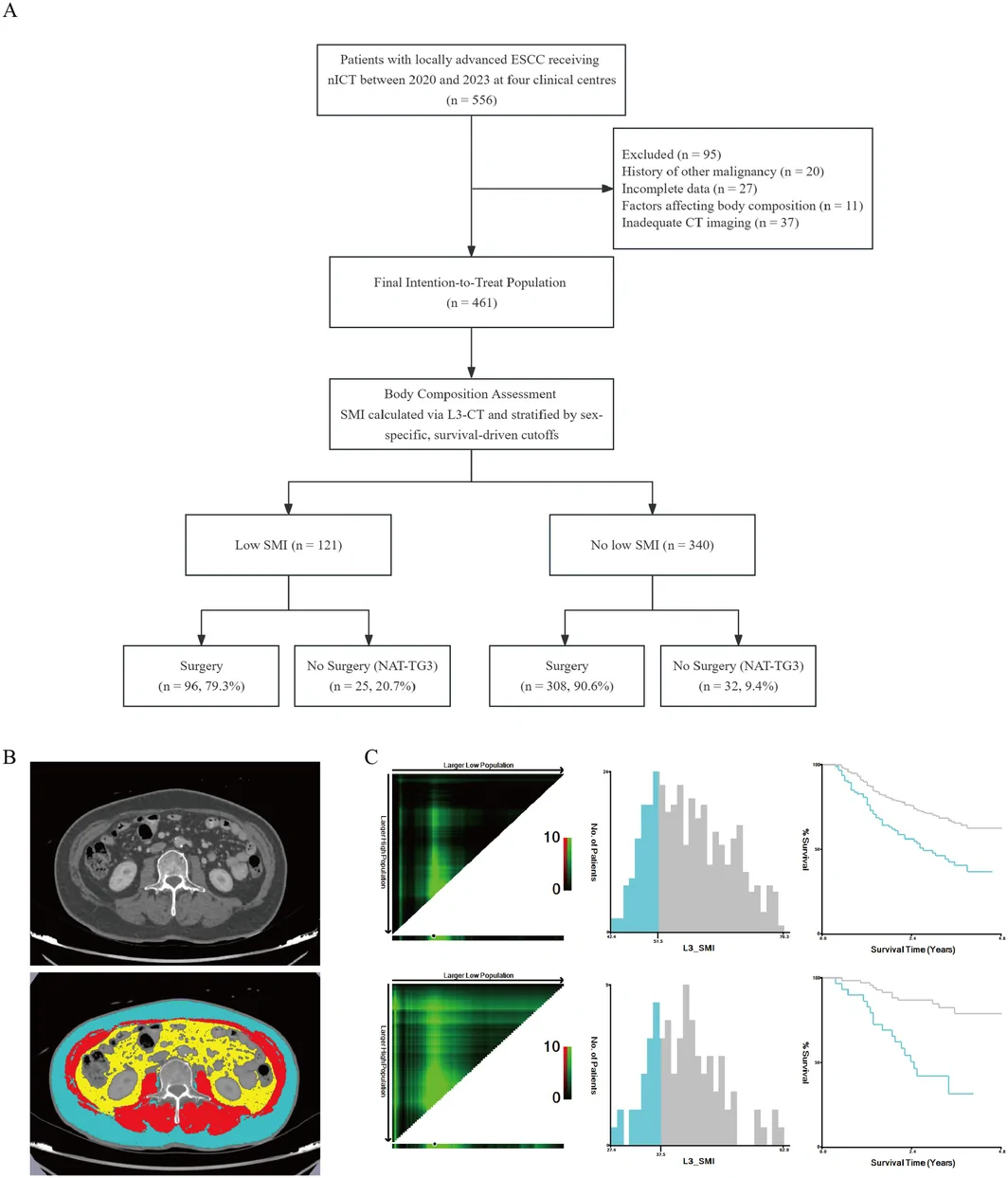
Methodological framework and identification of low SMI. (A) Study flow chart illustrating patient screening, inclusion, and group allocation. (B) Representative CT‐based body‐composition segmentation at the L3 level. Skeletal muscle is shown in red, visceral adipose tissue in yellow and subcutaneous adipose tissue in blue. (C) X‐tile plots identifying the optimal sex‐specific SMI cutoff values for OS and the corresponding Kaplan–Meier survival curves for the low‐SMI and non‐low‐SMI groups. Log‐rank test showed significant differences in overall survival between groups (*p* < 0.001). Total number of events: 165 (Non‐low SMI = 98, Low SMI = 67). *Note:* CT, computed tomography; L3, third lumbar vertebra; OS, overall survival.

**TABLE 1:** Baseline characteristics of the total cohort stratified according to low‐SMI status.

Variables	Total (*n* = 461)	No low SMI (*n* = 340)	Low SMI (*n* = 121)	*p*
Age, mean ± SD (years)	61.16 ± 7.28	60.93 ± 7.17	61.82 ± 7.58	0.249
BMI, *n* (%)				< 0.001
< 18.5 kg/m^2^	65 (14.10)	35 (10.29)	30 (24.79)	
18.5–25 kg/m^2^	334 (72.45)	244 (71.76)	90 (74.38)	
≥ 25 kg/m^2^	62 (13.45)	61 (17.94)	1 (0.83)	
L3 SMA, cm^2^, mean ± SD	150.85 ± 30.88	158.88 ± 29.30	128.29 ± 23.14	< 0.001
L3 SMI, cm^2^/m^2^, mean ± SD	54.61 ± 9.86	57.91 ± 8.72	45.35 ± 6.38	< 0.001
Sex, *n* (%)				0.396
Male	363 (78.74)	271 (79.71)	92 (76.03)	
Female	98 (21.26)	69 (20.29)	29 (23.97)	
Smoking, *n* (%)				0.916
No	200 (43.38)	148 (43.53)	52 (42.98)	
Yes	261 (56.62)	192 (56.47)	69 (57.02)	
Drinking, *n* (%)				0.888
No	268 (58.13)	197 (57.94)	71 (58.68)	
Yes	193 (41.87)	143 (42.06)	50 (41.32)	
Hypertension, *n* (%)				0.786
No	407 (88.29)	301 (88.53)	106 (87.60)	
Yes	54 (11.71)	39 (11.47)	15 (12.40)	
Diabetes, *n* (%)				0.887
No	437 (94.79)	322 (94.71)	115 (95.04)	
Yes	24 (5.21)	18 (5.29)	6 (4.96)	
ECOG, *n* (%)				0.284
ECOG 0	202 (43.82)	154 (45.29)	48 (39.67)	
ECOG ≥ 1	259 (56.18)	186 (54.71)	73 (60.33)	
Tumour location, *n* (%)				0.128
Upper	69 (14.97)	57 (16.76)	12 (9.92)	
Middle	265 (57.48)	195 (57.35)	70 (57.85)	
Lower	127 (27.55)	88 (25.88)	39 (32.23)	
Tumour size, *n* (%)				0.332
> 5 cm	227 (49.24)	172 (50.59)	55 (45.45)	
≤ 5 cm	234 (50.76)	168 (49.41)	66 (54.55)	
cT stage, *n* (%)				0.132
1	12 (2.60)	9 (2.65)	3 (2.48)	
2	100 (21.69)	81 (23.82)	19 (15.70)	
3	238 (51.63)	176 (51.76)	62 (51.24)	
4	111 (24.08)	74 (21.76)	37 (30.58)	
cN stage, *n* (%)				0.638
0	82 (17.79)	63 (18.53)	19 (15.70)	
1	121 (26.25)	91 (26.76)	30 (24.79)	
2	187 (40.56)	132 (38.82)	55 (45.45)	
3	71 (15.40)	54 (15.88)	17 (14.05)	
cTNM stage, *n* (%)				0.109
II	84 (18.22)	67 (19.71)	17 (14.05)	
III	220 (47.72)	166 (48.82)	54 (44.63)	
IVA	157 (34.06)	107 (31.47)	50 (41.32)	
Immunotherapy, *n* (%)				0.390
Nivolumab	40 (8.68)	27 (7.94)	13 (10.74)	
Pembrolizumab	75 (16.27)	54 (15.88)	21 (17.36)	
Camrelizumab	204 (44.25)	149 (43.82)	55 (45.45)	
Tislelizumab	63 (13.67)	45 (13.24)	18 (14.88)	
Sintilimab	79 (17.14)	65 (19.12)	14 (11.57)	
Cycle, *n* (%)				0.027
2–4 cycles	416 (90.24)	313 (92.06)	103 (85.12)	
1 cycle	45 (9.76)	27 (7.94)	18 (14.88)	
Blood inflammatory markers				
Albumin, mean ± SD	40.83 ± 3.91	41.09 ± 3.65	40.11 ± 4.50	0.018
Platelet count, mean ± SD	259.13 ± 69.92	251.80 ± 69.30	279.71 ± 67.78	< 0.001
Neutrophil count, mean ± SD	4.37 ± 1.69	4.31 ± 1.73	4.55 ± 1.56	0.173
Lymphocyte count, mean ± SD	1.93 ± 0.60	1.94 ± 0.58	1.92 ± 0.65	0.852
NLR, mean ± SD	2.45 ± 1.18	2.39 ± 1.14	2.63 ± 1.29	0.054
PLR, mean ± SD	145.45 ± 56.41	139.74 ± 51.38	161.51 ± 66.26	0.001
SII, mean ± SD	643.20 ± 382.70	611.88 ± 371.75	731.20 ± 400.51	0.003
NPR, mean ± SD	66.00 ± 26.47	64.80 ± 24.00	69.34 ± 32.31	0.105

*Note:* Categorical variables are presented as *n* (%). Continuous variables are presented as mean ± standard deviation or median (interquartile range), as appropriate. Low SMI was defined using sex‐specific L3 skeletal muscle index (SMI) cutoff values.

Abbreviations: BMI, body mass index; cTNM, clinical tumour–node–metastasis stage; ECOG PS, Eastern Cooperative Oncology Group performance status; NLR, neutrophil‐to‐lymphocyte ratio; NPR, neutrophil‐to‐platelet ratio; PLR, platelet‐to‐lymphocyte ratio; SII, systemic immune‐inflammation index; SMA, skeletal muscle area; SMI, skeletal muscle index.

Baseline age and sex distributions did not differ significantly between patients with and without low SMI. Low SMI was associated with a significantly higher prevalence of BMI < 18.5 kg/m^2^ (24.8% vs. 10.3%, *p* < 0.001), although most patients with low SMI had a BMI ≥ 18.5 kg/m^2^, highlighting the discordance between BMI and skeletal muscle quantity (Figure S1). Patients with low SMI also exhibited lower serum albumin levels and higher platelet‐to‐lymphocyte ratio and systemic immune‐inflammation index values (Table 1).

### 3.2 Low SMI, Neoadjuvant Treatment Tolerance and Toxicity

Low SMI was associated with a less favourable NAT‐TG distribution (Figure 2A). NAT‐TG1 was achieved in 72.9% of patients without low SMI compared with 48.8% of patients with low SMI, whereas NAT‐TG3 occurred more frequently in the low‐SMI group (20.7% vs. 9.4%; *p* < 0.001). Survival declined progressively across NAT‐TG categories. The 2‐year OS rates were 85.7%, 78.4% and 28.1% for NAT‐TG1, NAT‐TG2 and NAT‐TG3, respectively, and the corresponding 3‐year OS rates were 78.2%, 64.2% and 18.0% (*p* < 0.001; Figure 2B). Detailed survival estimates with 95% confidence intervals are provided in Table S2. The Sankey diagrams further illustrated the longitudinal treatment and survival trajectories across NAT‐TG categories (Figure 2C). Among the 57 patients classified as NAT‐TG3, the dominant reason for surgical omission was severe treatment‐related toxicity in 30 patients (52.6%), persistent clinical or functional deterioration in 21 (36.8%) and disease progression in 6 (10.5%) (Table S3).

**FIGURE 2:**
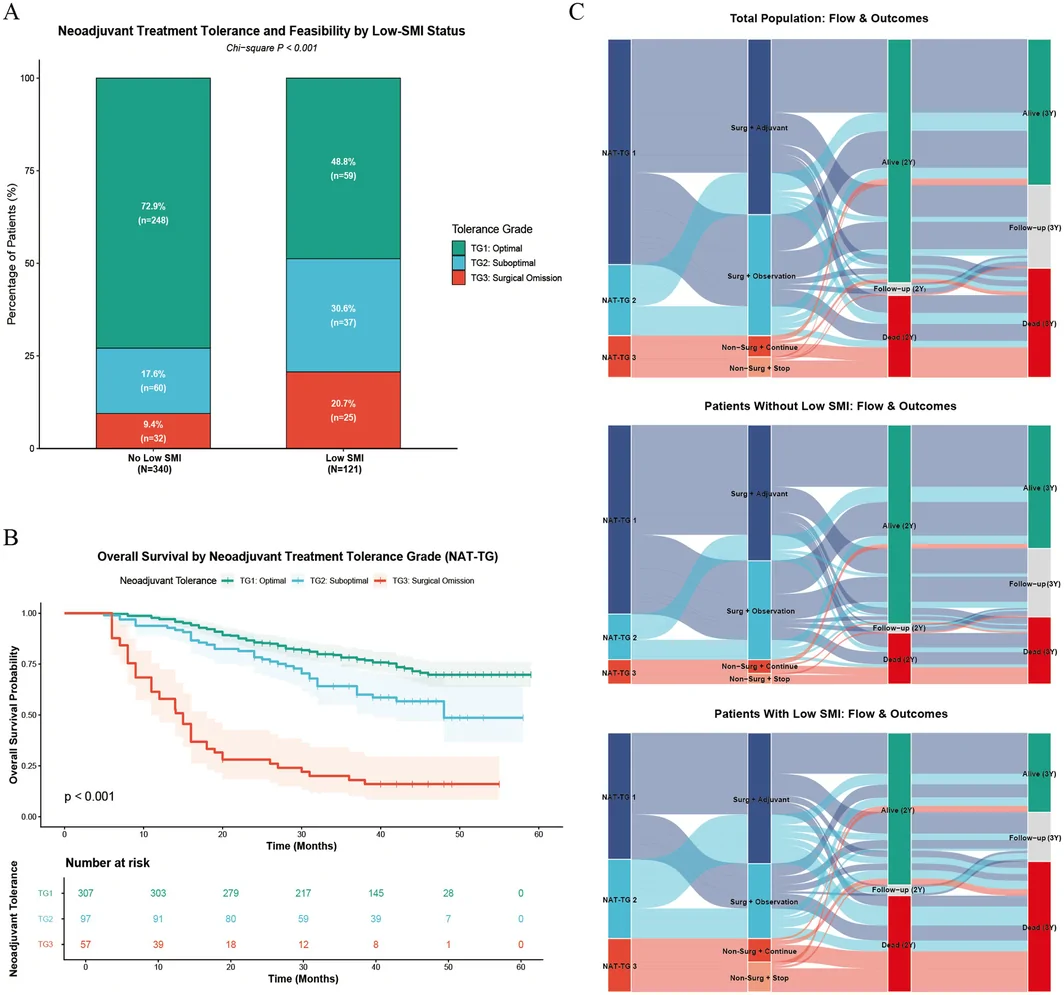
Impact of low SMI on neoadjuvant treatment tolerance and longitudinal patient trajectories. (A) Distribution of neoadjuvant treatment tolerance grades in patients with and without low SMI (Chi‐square *p* < 0.001). (B) Kaplan–Meier curves for overall survival in the total cohort stratified by NAT‐TG. Log‐rank test showed a stepwise decline in survival from Grade 1 to Grade 3 (*p* < 0.001), with 2‐year OS rates of 85.7%, 78.4% and 28.1% and corresponding 3‐year OS rates of 78.2%, 64.2% and 18.0% for NAT‐TG1, NAT‐TG2 and NAT‐TG3, respectively. Total number of events: 165 (TG1 = 78, TG2 = 40, TG3 = 47). (C) Sankey diagrams illustrating the clinical pathways for the total cohort (top), non‐low‐SMI group (middle) and low‐SMI group (bottom). *Note:* For the Sankey diagrams, the columns represent (from left to right): (1) Neoadjuvant treatment tolerance grade; (2) Post‐neoadjuvant treatment pathway, including surgical patients (with or without adjuvant immunotherapy) and non‐surgical patients (continued treatment or cessation); (3) 2‐year survival status; and (4) 3‐year survival status.

The overall distribution of treatment‐related adverse event severity differed between patients with and without low SMI (*p* = 0.012; Table 2), with grade ≥ 3 events occurring in 36.4% and 22.9%, respectively. Differences were particularly evident for gastrointestinal and hematologic toxicities (Figure S2). In multivariable logistic regression, low SMI remained independently associated with an unfavourable NAT‐TG (NAT‐TG2–3 vs. NAT‐TG1) after adjustment for BMI and clinical T stage (adjusted OR, 2.48; 95% CI, 1.62–3.82; *p* < 0.001; Table 3). Advanced clinical T stage was also independently associated with an unfavourable NAT‐TG. After excluding the six patients whose surgical omission was primarily attributable to disease progression, the association between low SMI and the restricted tolerance/feasibility endpoint remained materially unchanged (adjusted OR, 2.51; 95% CI, 1.59–3.95; *p* < 0.001; Table S4).

**TABLE 2:** Comparison of treatment‐related adverse events (TRAEs) during neoadjuvant immunochemotherapy between patients with and without low SMI.

Variables	Total (*n* = 461)	No low SMI (*n* = 340)	Low SMI (*n* = 121)	*p*
Any grade TRAEs, *n* (%)				0.012
0	128 (27.77)	102 (30.00)	26 (21.49)	
1–2	211 (45.77)	160 (47.06)	51 (42.15)	
≥ 3	122 (26.46)	78 (22.94)	44 (36.36)	
Skin toxicities, *n* (%)				0.125
0	362 (78.52)	260 (76.47)	102 (84.30)	
1–2	80 (17.35)	63 (18.53)	17 (14.05)	
≥ 3	19 (4.12)	17 (5.00)	2 (1.65)	
Gastrointestinal toxicities, *n* (%)				0.020
0	347 (75.27)	267 (78.53)	80 (66.12)	
1–2	87 (18.87)	57 (16.76)	30 (24.79)	
≥ 3	27 (5.86)	16 (4.71)	11 (9.09)	
Lung toxicities, *n* (%)				0.899
0	444 (96.31)	328 (96.47)	116 (95.87)	
1–2	14 (3.04)	10 (2.94)	4 (3.31)	
≥ 3	3 (0.65)	2 (0.59)	1 (0.83)	
Endocrine toxicity, *n* (%)				0.722
0	415 (90.02)	306 (90.00)	109 (90.08)	
1–2	38 (8.24)	29 (8.53)	9 (7.44)	
≥ 3	8 (1.74)	5 (1.47)	3 (2.48)	
Renal toxicity, *n* (%)				0.362
0	447 (96.96)	331 (97.35)	116 (95.87)	
1–2	12 (2.60)	7 (2.06)	5 (4.13)	
≥ 3	2 (0.43)	2 (0.59)	0 (0.00)	
Nervous system toxicities, *n* (%)				1.000
0	455 (98.70)	336 (98.82)	119 (98.35)	
1–2	6 (1.30)	4 (1.18)	2 (1.65)	
Hematologic toxicities, *n* (%)				0.006
0	226 (49.02)	177 (52.06)	49 (40.50)	
1–2	146 (31.67)	109 (32.06)	37 (30.58)	
≥ 3	89 (19.31)	54 (15.88)	35 (28.93)	
Cardiovascular toxicities, *n* (%)				1.000
0	459 (99.57)	338 (99.41)	121 (100.00)	
1–2	2 (0.43)	2 (0.59)	0 (0.00)	

*Note:* Adverse events were graded according to the Common Terminology Criteria for Adverse Events (CTCAE) version 5.0. *p* values reflect the overall comparison of toxicity‐grade distributions (Grade 0, Grade 1–2 and Grade ≥ 3) between groups.

**TABLE 3:** Univariable and multivariable logistic regression analyses of unfavourable NAT‐TG (NAT‐TG2–3 vs. NAT‐TG1).

Variable	Univariable analysis		Multivariable analysis	
	OR (95% CI)	*p*	OR (95% CI)	*p*
Sex				
Male	1.00 (Reference)			
Female	0.99 (0.62–1.58)	0.973		
ECOG				
ECOG 0	1.00 (Reference)			
ECOG ≥ 1	1.23 (0.83–1.81)	0.297		
BMI				
< 18.5 kg/m^2^	1.00 (Reference)		1.00 (Reference)	
18.5–25 kg/m^2^	0.62 (0.38–1.03)	0.066	0.67 (0.40–1.13)	0.135
≥ 25 kg/m^2^	0.41 (0.19–0.87)	0.019	0.60 (0.27–1.33)	0.209
Low SMI				
No	1.00 (Reference)		1.00 (Reference)	
Yes	2.76 (1.82–4.16)	< 0.001	2.48 (1.62–3.82)	< 0.001
Age (years)				
≤ 60	1.00 (Reference)			
> 60	1.40 (0.95–2.05)	0.087		
Smoking				
No	1.00 (Reference)			
Yes	1.30 (0.89–1.92)	0.179		
Drinking				
No	1.00 (Reference)			
Yes	0.77 (0.52–1.13)	0.179		
Hypertension				
No	1.00 (Reference)			
Yes	1.21 (0.68–2.16)	0.514		
Diabetes				
No	1.00 (Reference)			
Yes	1.16 (0.59–2.27)	0.662		
Tumour location				
Upper	1.00 (Reference)			
Middle	0.74 (0.43–1.28)	0.282		
Lower	0.74 (0.40–1.35)	0.320		
Tumour size				
> 5 cm	1.00 (Reference)			
≤ 5 cm	1.12 (0.77–1.64)	0.555		
cT stage				
T 1–2	1.00 (Reference)		1.00 (Reference)	
T 3–4	2.11 (1.30–3.45)	0.003	2.05 (1.24–3.38)	0.005
cN stage				
N 0	1.00 (Reference)			
N +	1.48 (0.87–2.50)	0.146		

Abbreviations: BMI, body mass index; CI, confidence interval; ECOG, Eastern Cooperative Oncology Group; NAT‐TG, neoadjuvant treatment tolerance grade; OR, odds ratio; SMI, skeletal muscle index.

### 3.3 Postoperative Complications in the Surgical Cohort

Among the 404 patients who underwent oesophagectomy (Table S5), NAT‐TG2 occurred more frequently among patients with low SMI than among those without low SMI (38.5% vs. 19.5%; *p* < 0.001). The overall Clavien–Dindo complication distribution differed between groups (*p* = 0.002), with grade ≥III complications occurring in 22.9% of patients with low SMI and 11.0% of those without low SMI. Pneumonia and pleural effusion severity distributions also differed between groups (*p* = 0.006 and *p* = 0.007, respectively); patients with low SMI had higher any‐grade rates of pneumonia (38.5% vs. 26.0%) and pleural effusion (52.1% vs. 37.3%). ICU admission was numerically more frequent in patients with low SMI (9.4% vs. 4.6%; *p* = 0.075), whereas 30‐day readmission rates were similar between groups (4.2% vs. 3.2%; *p* = 0.912). No deaths occurred within 30 days after surgery. Differences in anastomotic leakage, cardiac arrhythmia and incisional complications were not statistically significant (Table S6).

In the multivariable analysis adjusting for age, sex, BMI, serum albumin, operative approach, lymphadenectomy extent, tumour location and participating centre, low SMI remained independently associated with severe postoperative complications (adjusted OR, 2.92; 95% CI, 1.51–5.63; *p* = 0.001; Table S7).

### 3.4 Survival Outcomes and Prognostic Factors

During a median follow‐up of 43 months, 165 deaths occurred in the ITT population, and the 1‐year mortality rate was 8.5% (95% CI, 5.9%–11.0%). Low SMI was significantly associated with poorer OS in the ITT population (Figure 3A). Median OS was not reached in patients without low SMI and was 35.0 months in patients with low SMI. The 2‐ and 3‐year OS rates were 80.9% and 74.4% in patients without low SMI, compared with 66.1% and 49.4% in patients with low SMI, respectively. Detailed 2‐ and 3‐year survival estimates across the ITT and surgical populations are summarized in Table S2.

**FIGURE 3:**
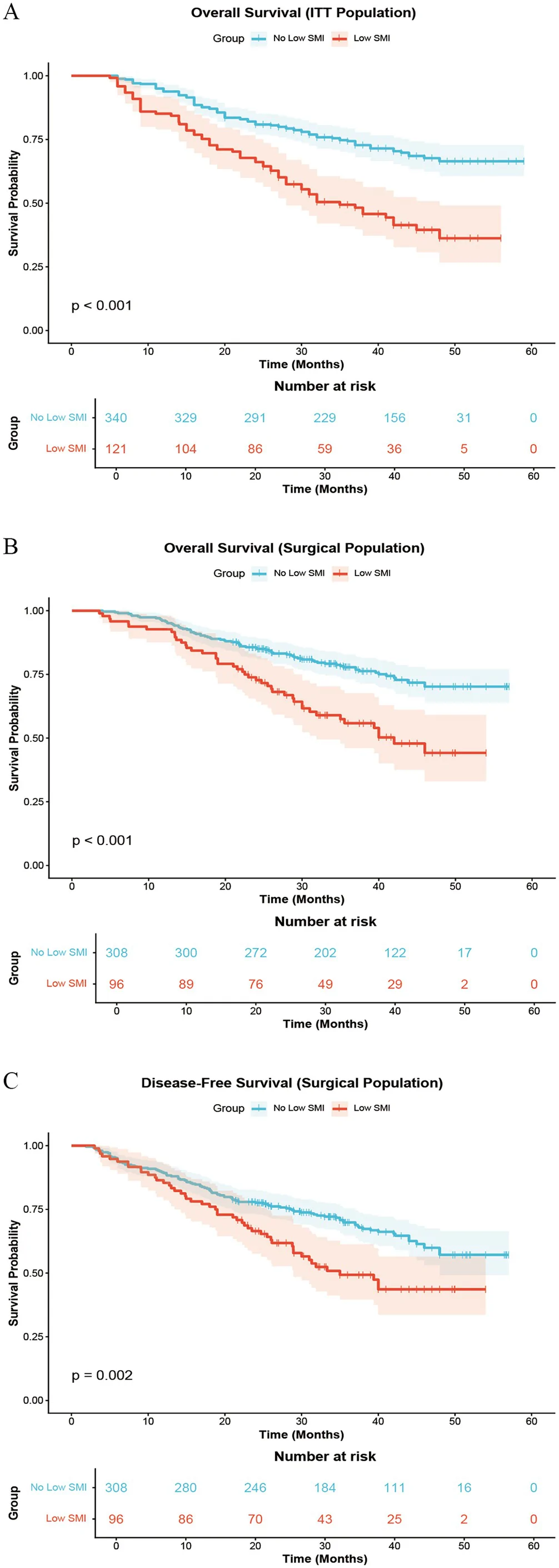
Survival outcomes stratified according to Low‐SMI status. (A) Overall survival in the total intention‐to‐treat population. Log‐rank test showed *p* < 0.001. Univariable Cox regression showed an HR of 2.38 (95% CI, 1.74–3.25; *p* < 0.001) for patients with low SMI versus those without low SMI. Total number of events: 165 (No low SMI = 98, Low SMI = 67). (B) Overall survival in the surgical population (*n* = 404). Multivariable Cox regression yielded an adjusted HR of 1.91 (95% CI, 1.31–2.80; *p* < 0.001) for low SMI. Total number of events: 118 (No low SMI = 74, Low SMI = 44). (C) Disease‐free survival in the surgical population (*n* = 404). Multivariable Cox regression yielded an adjusted HR of 1.57 (95% CI, 1.11–2.22; *p* = 0.011) for low SMI. Total number of events: 150 (No low SMI = 102, Low SMI = 48).

In the primary clinically specified ITT model, low SMI remained independently associated with poorer OS (adjusted HR, 2.36; 95% CI, 1.67–3.33; *p* < 0.001). Similar estimates were obtained in the data‐driven Model 1 (adjusted HR, 2.17; 95% CI, 1.56–3.00; *p* < 0.001) and the sensitivity model additionally adjusted for actual nICT cycles and PD‐1 inhibitor type (adjusted HR, 2.38; 95% CI, 1.68–3.38; *p* < 0.001; Table S8). Neither treatment year, planned cycle protocol, PD‐1 inhibitor type, nor taxane or platinum backbone was significantly associated with OS in univariable analyses. Receipt of only one nICT cycle was associated with poorer OS in univariable analysis (HR, 1.68; 95% CI, 1.08–2.64; *p* = 0.023), although the association was attenuated after multivariable adjustment in Model 3 (adjusted HR, 1.57; 95% CI, 0.98–2.53; *p* = 0.061; Tables S8 and S9).

Similar findings were observed in the surgical population. The 2‐ and 3‐year OS rates were 85.3% and 77.8% in patients without low SMI and 72.8% and 55.8% in patients with low SMI, respectively. The corresponding DFS rates were 77.6% and 69.9% versus 66.5% and 49.3% (Figure 3B,C). On multivariable analysis, low SMI remained independently associated with poorer OS (adjusted HR, 1.91; 95% CI, 1.31–2.80; *p* < 0.001) and DFS (adjusted HR, 1.57; 95% CI, 1.11–2.22; *p* = 0.011; Table 4).

**TABLE 4:** Univariable and multivariable Cox proportional hazards analyses for overall and disease‐free survival in the surgical population.

Variables	OS					DFS				
		Univariable analysis		Multivariable analysis			Univariable analysis		Multivariable analysis	
		HR (95% CI)	*p*	HR (95% CI)	*p*		HR (95% CI)	*p*	HR (95% CI)	*p*
Sex										
Male		1.00 (Reference)					1.00 (Reference)			
Female		0.76 (0.47–1.22)	0.251				0.94 (0.63–1.40)	0.760		
Age										
≤ 60		1.00 (Reference)					1.00 (Reference)			
> 60		1.17 (0.82–1.69)	0.391				1.13 (0.82–1.56)	0.453		
ECOG										
ECOG 0		1.00 (Reference)					1.00 (Reference)			
ECOG ≥ 1		1.09 (0.80–1.49)	0.568				1.09 (0.82–1.44)	0.565		
BMI										
< 18.5		1.00 (Reference)					1.00 (Reference)			
18.5–25		0.96 (0.57–1.61)	0.872				1.05 (0.65–1.69)	0.855		
≥ 25		0.96 (0.48–1.93)	0.914				1.31 (0.72–2.39)	0.371		
Low SMI										
No		1.00 (Reference)		1.00 (Reference)			1.00 (Reference)		1.00 (Reference)	
Yes		2.21 (1.52–3.20)	< 0.001	1.91 (1.31–2.80)	< 0.001		1.71 (1.22–2.42)	0.002	1.57 (1.11–2.22)	0.011
Adjuvant immunotherapy										
No		1.00 (Reference)					1.00 (Reference)			
Yes		0.76 (0.53–1.09)	0.139				1.00 (0.72–1.38)	0.982		
TRG										
TRG 0–1		1.00 (Reference)		1.00 (Reference)			1.00 (Reference)		1.00 (Reference)	
TRG 2–3		3.32 (2.10–5.23)	< 0.001	2.12 (1.27–3.56)	0.004		2.17 (1.52–3.11)	< 0.001	1.46 (0.97–2.22)	0.072
Smoking										
No		1.00 (Reference)					1.00 (Reference)			
Yes		1.04 (0.72–1.49)	0.849				1.02 (0.74–1.40)	0.917		
Drinking										
No		1.00 (Reference)					1.00 (Reference)			
Yes		1.22 (0.85–1.75)	0.286				1.16 (0.84–1.60)	0.360		
Hypertension										
No		1.00 (Reference)					1.00 (Reference)			
Yes		0.81 (0.43–1.50)	0.495				0.97 (0.59–1.61)	0.907		
Diabetes										
No		1.00 (Reference)					1.00 (Reference)			
Yes		0.93 (0.49–1.78)	0.828				0.92 (0.52–1.63)	0.776		
Tumour location										
Upper		1.00 (Reference)					1.00 (Reference)			
Middle		1.31 (0.72–2.36)	0.376				1.05 (0.65–1.71)	0.837		
Lower		1.42 (0.75–2.67)	0.281				1.11 (0.66–1.89)	0.688		
Tumour size										
≤ 5 cm		1.00 (Reference)		1.00 (Reference)			1.00 (Reference)			
> 5 cm		1.56 (1.08–2.25)	0.018	1.35 (0.93–1.96)	0.120		1.31 (0.95–1.80)	0.106		
Differentiation										
G1		1.00 (Reference)		1.00 (Reference)			1.00 (Reference)		1.00 (Reference)	
G2		1.36 (0.86–2.16)	0.192	1.28 (0.80–2.03)	0.305		1.20 (0.81–1.78)	0.351	1.12 (0.75–1.65)	0.582
G3		2.20 (1.32–3.67)	0.002	1.72 (1.02–2.90)	0.042		1.73 (1.10–2.71)	0.017	1.39 (0.88–2.21)	0.155
ypTNM stage										
I		1.00 (Reference)		1.00 (Reference)			1.00 (Reference)		1.00 (Reference)	
II		1.64 (0.83–3.23)	0.152	0.94 (0.46–1.95)	0.876		1.06 (0.58–1.95)	0.840	0.81 (0.42–1.54)	0.514
III		3.16 (1.90–5.24)	< 0.001	1.83 (1.04–3.22)	0.035		2.58 (1.72–3.89)	< 0.001	1.93 (1.22–3.05)	0.005
IVA		7.83 (3.86–15.88)	< 0.001	4.50 (2.11–9.58)	< 0.001		5.96 (3.16–11.24)	< 0.001	4.16 (2.09–8.26)	< 0.001

Abbreviations: BMI, body mass index; CI, confidence interval; DFS, disease‐free survival; ECOG, Eastern Cooperative Oncology Group; HR, hazard ratio; OS, overall survival; SMI, skeletal muscle index; TRG, tumour regression grade; ypTNM, post‐neoadjuvant pathological tumour–node–metastasis stage.

### 3.5 Sensitivity Analyses and Internal Model Validation

The associations were generally consistent when low SMI was defined using published thresholds (Table S10). Under the Prado definition, low SMI remained associated with an unfavourable NAT‐TG (NAT‐TG2–3 vs. NAT‐TG1, adjusted OR, 2.44; 95% CI, 1.57–3.78; *p* < 0.001), ITT OS in Models 2 and 3 (adjusted HRs, 1.98 and 1.94, respectively; both *p* < 0.001) and surgical OS (adjusted HR, 1.65; 95% CI, 1.13–2.41; *p* = 0.010). Under the broader Fearon definition, the associations with unfavourable NAT‐TG and ITT OS remained statistically significant, whereas the association with surgical OS was attenuated (adjusted HR, 1.37; 95% CI, 0.95–1.99; *p* = 0.091).

Centre‐stratified bootstrap internal validation of the fitted Cox models (1000 resamples) yielded apparent and optimism‐corrected C‐indices of 0.711 and 0.686 for Model 2, and 0.716 and 0.682 for Model 3, respectively. Kaplan–Meier‐based calibration curves demonstrated adequate agreement between predicted and observed 3‐year OS probabilities (Figure S3).

### 3.6 Exploratory Association of Adjuvant Immunotherapy With Survival

Among patients undergoing surgery, 59.2% received postoperative adjuvant immunotherapy, with comparable use between patients with and without low SMI (Table S11). No clear survival association was observed in patients without low SMI (Figure S4). Among patients with low SMI, adjuvant immunotherapy was associated with higher 3‐year OS (63.8% vs. 43.1%; *p* = 0.033) and DFS (59.0% vs. 34.5%; *p* = 0.027) compared with observation (Figures S5A and S5B).

After adjustment for age, sex, ypTNM stage and postoperative Clavien–Dindo grade within the low‐SMI subgroup, adjuvant immunotherapy remained associated with lower hazards of death (adjusted HR, 0.47; 95% CI, 0.25–0.88; *p* = 0.019) and recurrence or death (adjusted HR, 0.47; 95% CI, 0.26–0.87; *p* = 0.016; Table S12). In the 6‐month landmark analysis, the effect estimates remained directionally similar, although the confidence intervals crossed the null for OS (HR, 0.58; 95% CI, 0.31–1.09; *p* = 0.089) and DFS (HR, 0.56; 95% CI, 0.31–1.02; *p* = 0.052; Figures S5C and S5D). These findings should therefore be considered exploratory.

## 4 Discussion

Using multicentre real‐world data, this study evaluated pretreatment CT‐derived SMI as a host‐related marker in patients with locally advanced OSCC receiving nICT. Low SMI was independently associated with less favourable neoadjuvant treatment tolerance and feasibility, severe postoperative complications and poorer long‐term outcomes. We further introduced NAT‐TG as a framework for characterizing treatment tolerance and feasibility across the neoadjuvant‐to‐surgery pathway. The principal associations remained consistent after broader multivariable adjustment, exclusion of progression‐related surgical omissions and application of published SMI thresholds.

Although nICT followed by surgery has emerged as a promising treatment strategy for locally advanced OSCC, a clinically relevant proportion of patients require treatment modification or fail to complete the planned neoadjuvant‐to‐surgery pathway because of toxicity, functional deterioration or disease progression [21]. Treatment‐related adverse events may also delay surgery beyond the optimal therapeutic window, which has been shown to adversely affect DFS and long‐term outcomes [22, 23]. NAT‐TG was therefore designed as an integrated measure of treatment tolerance and feasibility, incorporating dose reduction, treatment delay, premature discontinuation and failure to undergo curative surgery. In our cohort, 21.0% of patients required treatment modification (NAT‐TG2), whereas 12.4% experienced unplanned failure to undergo curative surgery (NAT‐TG3). Among the 57 NAT‐TG3 patients, 30 failed to undergo surgery predominantly because of severe treatment‐related toxicity, 21 because of persistent clinical or functional deterioration and six because of disease progression despite otherwise acceptable treatment tolerance. After excluding these six cases, the association persisted for the restricted endpoint. These findings support NAT‐TG as a composite measure of treatment tolerance and feasibility rather than physiological reserve alone.

The prevalence of low SMI in our cohort (26.2%) was consistent with that reported in previous Asian CT‐based studies and highlighted the frequent dissociation between BMI and skeletal muscle mass [11, 24]. Most patients with low SMI had a normal or even elevated BMI, indicating that conventional anthropometric measures alone may fail to identify reduced skeletal muscle reserve (Figure S1). This discordance supports the potential value of CT‐based body‐composition assessment for identifying patients whose physiological vulnerability may not be apparent from BMI alone [25, 26]. The association with unfavourable NAT‐TG was also maintained under the published Prado and Fearon SMI thresholds [18, 20], although the prevalence of low SMI and the magnitude of the survival associations varied across definitions. Several mechanisms may contribute to impaired treatment tolerance in patients with low muscle mass. Skeletal muscle depletion may alter drug distribution and clearance, potentially increasing systemic exposure to cytotoxic agents [27, 28]. Moreover, low skeletal muscle mass has been associated with chronic inflammation, metabolic dysregulation and immunosenescence, which may increase susceptibility to chemotherapy‐ and immune‐related toxicities [29]. Consistent with these potential mechanisms, patients with low SMI in our cohort experienced higher crude rates of severe hematologic and gastrointestinal toxicities. Nevertheless, these mechanistic explanations remain inferential and require prospective evaluation.

The association of low SMI with adverse outcomes extended beyond the neoadjuvant period. Even among patients who underwent curative resection, those with low SMI were more likely to have required treatment modification and experienced a higher incidence of severe postoperative complications, particularly pneumonia and pleural effusion. The association with severe complications persisted after adjustment for age, sex, BMI, serum albumin, operative approach, lymphadenectomy extent, tumour location and participating centre. These findings are consistent with previous systematic reviews and cohort studies linking reduced SMI to postoperative pulmonary morbidity after oesophagectomy [30]. Potential explanations include reduced respiratory muscle strength, impaired cough effectiveness and systemic inflammatory or metabolic vulnerability [31, 32]. However, the present observational data cannot determine whether muscle depletion directly contributes to these complications or serves as a marker of broader physiological vulnerability.

Skeletal muscle depletion has been increasingly recognized as a prognostic determinant in oesophageal cancer across treatment contexts [33]. A meta‐analysis of 21 studies reported that low SMI was associated with poorer OS and DFS in resectable oesophageal cancer, with pooled hazard ratios of 1.56 and 1.73, respectively [34]. Similar associations have been reported in unresectable disease and among patients experiencing skeletal muscle loss during neoadjuvant treatment [14, 35]. Our findings extend this evidence to patients receiving nICT and, importantly, to an ITT population that included patients who did not proceed to surgery. The association between low SMI and OS remained stable across three centre‐stratified multivariable models. Bootstrap internal validation of Models 2 and 3, with the study‐derived SMI cutoff held fixed, indicated reasonably stable model performance after optimism correction. The moderate C‐indices nevertheless suggest that these models should not be interpreted as stand‐alone clinical prediction tools. Analyses using published SMI thresholds generally supported the direction of the primary findings, although attenuation of the surgical OS association under the broader Fearon definition indicates that the estimated prognostic magnitude remains partly dependent on the selected threshold.

An exploratory association between postoperative adjuvant immunotherapy and survival outcomes was observed among patients with low SMI, whereas no clear association was identified among patients without low SMI. Within the low‐SMI subgroup, this association persisted after adjustment for age, sex, ypTNM stage and postoperative complication severity. In the 6‐month landmark analysis, the effect estimates remained directionally consistent for both OS and DFS, although the confidence intervals crossed the null. Taken together, the consistent direction across analyses supports this hypothesis‐generating association, although the landmark estimates were less precise and some residual confounding may remain. The CheckMate‐577 trial established adjuvant immunotherapy as standard care for selected patients, and emerging real‐world evidence supports its benefit in oesophageal squamous cell carcinoma [36, 37]. However, its role after nICT remains less clearly defined. Our findings suggest that reduced skeletal muscle reserve does not necessarily preclude a favourable association with postoperative immunotherapy. Because patients with low SMI more often experienced modified or incomplete neoadjuvant treatment delivery, they may remain at greater risk of residual disease. Postoperative immunotherapy may therefore provide additional disease control and partly offset the adverse impact of suboptimal neoadjuvant treatment intensity. This hypothesis requires prospective validation.

This study has several limitations. Its retrospective multicentre design may have introduced selection bias, treatment heterogeneity and residual confounding despite adjustment for clinical and centre‐level factors. The primary sex‐specific SMI cutoffs were derived from OS within the same cohort and may therefore overestimate prognostic performance. Bootstrap correction addressed optimism in the fitted models, while sensitivity analyses using published Prado and Fearon thresholds generally supported the direction of the findings; however, independent external validation of the study‐derived cutoffs was not available in the present study. NAT‐TG is a newly proposed composite measure, and retrospective classification of the dominant reason for failure to undergo surgery may be imperfect; nevertheless, the association remained after progression‐related cases were excluded. The adjuvant immunotherapy analysis was exploratory and remained susceptible to treatment‐selection and healthy‐survivor biases despite multivariable and landmark analyses. Muscle assessment relied on a single pretreatment CT scan without measures of muscle strength or physical performance; therefore, the present study evaluated low SMI rather than clinically diagnosed sarcopenia. These findings therefore require validation in prospective cohorts and in settings beyond experienced centres.

## 5 Conclusion

In conclusion, pretreatment low SMI was associated with less favourable neoadjuvant treatment tolerance and feasibility, a higher incidence of severe postoperative complications and poorer long‐term outcomes in patients with locally advanced OSCC receiving nICT. CT‐derived SMI may provide a practical adjunct for baseline risk stratification, but its role in guiding nutritional, physical or anticancer treatment interventions requires prospective validation.

## Funding

This work was supported by the Clinical Research Center for Thoracic Tumours of Fujian Province (grant number: 2024YGPT001) and Joint Funds for the Innovation of Science and Technology, Fujian Province (grant number: 2025Y9350).

## Ethics Statement

The study was conducted in accordance with the Declaration of Helsinki and was approved by the Institutional Review Board of Fujian Medical University Union Hospital (Approval No. 2025KY671), which waived individual informed consent because of the retrospective design. Data‐sharing permissions and local institutional authorizations were obtained from the other participating centres before centralized analysis of anonymized data. All authors of this manuscript comply with the guidelines of ethical authorship and publishing in the Journal of Cachexia, Sarcopenia and Muscle [38].

## Conflicts of Interest

The authors declare no conflicts of interest.

## Supporting information


**Table S1:** Treatment heterogeneity across participating clinical centres.
**Table S2:** Two‐ and three‐year survival estimates according to low‐SMI status and neoadjuvant treatment tolerance grade.
**Table S3:** Dominant reasons for surgical omission stratified by pretreatment low‐SMI status.
**Table S4:** Sensitivity analysis of baseline factors associated with the restricted neoadjuvant treatment tolerance/feasibility endpoint.
**Table S5:** Baseline characteristics of the surgical population stratified by low‐SMI status.
**Table S6:** Comparison of postoperative complications stratified by pretreatment low‐SMI status.
**Table S7:** Univariable and multivariable logistic regression analyses of predictors for severe postoperative complications.
**Table S8:** Univariable and multivariable Cox proportional hazards analyses for overall survival in the intention‐to‐treat population.
**Table S9:** Univariable Cox proportional hazards analysis of treatment‐related characteristics for overall survival.
**Table S10:** Sensitivity analyses using study‐derived and published definitions of low L3 skeletal muscle index.
**Table S11:** Comparison of baseline characteristics between patients receiving and not receiving adjuvant immunotherapy, stratified by low‐SMI status.
**Table S12:** Univariable and multivariable Cox proportional hazards analyses for overall survival and disease‐free survival within the low‐SMI subgroup.
**Figure S1:** Correlation and discordance between body mass index and skeletal muscle index.
**Figure S2:** Comparative profile of adverse events during neoadjuvant immunochemotherapy between groups.
**Figure S3:** Bootstrap calibration curves for 3‐year overall survival in the intention‐to‐treat population.
**Figure S4:** Subgroup analysis of the association between adjuvant immunotherapy and survival in the surgical population.
**Figure S5:** Exploratory association of adjuvant immunotherapy with survival in patients with low SMI.

## Data Availability

Raw patient‐level data are not publicly available because of institutional privacy requirements. De‐identified data and analytical R scripts may be obtained from the corresponding author upon reasonable academic request and approval by the participating institutions.
