# Supplementary material for: Low Skeletal Muscle Index Predicts Neoadjuvant Immunochemotherapy Tolerance and Survival in Oesophageal Squamous Cell Carcinoma
Source: J Cachexia Sarcopenia Muscle. 2026 Sep 30;17(5):e70392. doi: 10.1002/jcsm.70392 (PMC13627971; doi:10.1002/jcsm.70392)
Supplement: Supplementary file 1 [file JCSM-17-e70392-s001.docx]

**Supplementary Material**

*Low Skeletal Muscle Index Predicts Neoadjuvant Immunochemotherapy Tolerance and Survival in Esophageal Squamous Cell Carcinoma*

**Contents**

**Supplementary Tables**

**Table S1.** Treatment heterogeneity across participating clinical centers	2

**Table S2.** Two- and three-year survival estimates according to low-SMI status and neoadjuvant treatment tolerance grade	4

**Table S3.** Dominant reasons for surgical omission stratified by pretreatment low-SMI status	6

**Table S4.** Sensitivity analysis of baseline factors associated with the restricted neoadjuvant treatment tolerance/feasibility endpoint	7

**Table S5.** Baseline characteristics of the surgical population stratified by low-SMI status	9

**Table S6.** Comparison of postoperative complications stratified by pretreatment low-SMI status	12

**Table S7.** Univariable and multivariable logistic regression analyses of predictors for severe postoperative complications	14

**Table S8.** Univariable and multivariable Cox proportional hazards analyses for overall survival in the intention-to-treat population	16

**Table S9.** Univariable Cox proportional hazards analysis of treatment-related characteristics for overall survival	20

**Table S10.** Sensitivity analyses using study-derived and published definitions of low L3 skeletal muscle index	22

**Table S11.** Comparison of baseline characteristics between patients receiving and not receiving adjuvant immunotherapy, stratified by low-SMI status	23

**Table S12.** Univariable and multivariable Cox proportional hazards analyses for overall survival and disease-free survival within the low-SMI subgroup	28

**Supplementary Figures**

**Figure S1.** Correlation and discordance between body mass index and skeletal muscle index	30

**Figure S2.** Comparative profile of adverse events during neoadjuvant immunochemotherapy between groups	31

**Figure S3.** Bootstrap calibration curves for 3-year overall survival in the intention-to-treat population	32

**Figure S4.** Subgroup analysis of the association between adjuvant immunotherapy and survival in the surgical population	33

**Figure S5.** Exploratory association of adjuvant immunotherapy with survival in patients with low SMI	34
**Table S1. Treatment heterogeneity across participating clinical centers**

| **Characteristic** | **Overall (N = 461)** | **Center 1 (N = 230)** | **Center 2 (N = 87)** | **Center 3 (N = 83)** | **Center 4 (N = 61)** | ***P* value** |
| --- | --- | --- | --- | --- | --- | --- |
| **Year of Treatment, n (%)** | | | | | | 0.375 |
| 2020–2021 | 200 (43.38) | 93 (40.43) | 36 (41.38) | 40 (48.19) | 31 (50.82) |  |
| 2022–2023 | 261 (56.62) | 137 (59.57) | 51 (58.62) | 43 (51.81) | 30 (49.18) |  |
| **PD-1 Inhibitor, n (%)** | | | | | | 0.202 |
| Nivolumab | 40 (8.68) | 24 (10.43) | 10 (11.49) | 3 (3.61) | 3 (4.92) |  |
| Pembrolizumab | 75 (16.27) | 40 (17.39) | 13 (14.94) | 11 (13.25) | 11 (18.03) |  |
| Camrelizumab | 204 (44.25) | 95 (41.30) | 32 (36.78) | 41 (49.40) | 36 (59.02) |  |
| Tislelizumab | 63 (13.67) | 33 (14.35) | 14 (16.09) | 12 (14.46) | 4 (6.56) |  |
| Sintilimab | 79 (17.14) | 38 (16.52) | 18 (20.69) | 16 (19.28) | 7 (11.48) |  |
| **Taxane Backbone (Planned Dose), n (%)** | | | | | | 0.204 |
| Paclitaxel (135–175 mg/m²) | 304 (65.94) | 145 (63.04) | 54 (62.07) | 60 (72.29) | 45 (73.77) |  |
| Nab-paclitaxel (260 mg/m²) | 157 (34.06) | 85 (36.96) | 33 (37.93) | 23 (27.71) | 16 (26.23) |  |
| **Platinum Backbone (Planned Dose), n (%)** | | | | | | 0.583 |
| Cisplatin (75 mg/m²) | 246 (53.36) | 131 (56.96) | 44 (50.57) | 42 (50.60) | 29 (47.54) |  |
| Carboplatin (AUC 5) | 118 (25.60) | 51 (22.17) | 27 (31.03) | 24 (28.92) | 16 (26.23) |  |
| Nedaplatin (80 mg/m²) | 97 (21.04) | 48 (20.87) | 16 (18.39) | 17 (20.48) | 16 (26.23) |  |
| **Neoadjuvant Cycle Protocol, n (%)** | | | | | | — |
| Standard 2-cycle approach | 61 (13.23) | 0 (0.0) | 0 (0.0) | 0 (0.0) | 61 (100.0) |  |
| Standard 3-cycle approach | 400 (86.77) | 230 (100.0) | 87 (100.0) | 83 (100.0) | 0 (0.0) |  |
| **Actual Received Cycles, n (%)** | | | | | | 0.374 |
| ≥ 2 cycles | 416 (90.24) | 212 (92.17) | 76 (87.36) | 72 (86.75) | 56 (91.80) |  |
| 1 cycle (Early discontinuation) | 45 (9.76) | 18 (7.83) | 11 (12.64) | 11 (13.25) | 5 (8.20) |  |

Note: *P* values were calculated using the Pearson chi-square test or Fisher's exact test. Planned doses for chemotherapy backbones reflect standard institutional protocols. No P value was calculated for the planned-cycle protocol because it was institutionally predetermined and perfectly separated by center. Abbreviations: PD-1, programmed cell death protein 1; Nab-paclitaxel, nanoparticle albumin-bound paclitaxel.

**Table S2. Two- and three-year survival estimates according to low-SMI status and neoadjuvant treatment tolerance grade**

| **Population and outcome** | **Group** | **n** | **Median survival, months (95% CI)** | **2-year survival rate, % (95% CI)** | **3-year survival rate, % (95% CI)** |
| --- | --- | --- | --- | --- | --- |
| **Intention-to-treat population: OS** | NAT-TG1: Optimal tolerance | 307 | Not reached (not estimable) | 85.7 (81.8–89.7) | 78.2 (73.6–83.1) |
|  | NAT-TG2: Suboptimal tolerance | 97 | 48.0 (39.0–not estimable) | 78.4 (70.6–87.0) | 64.2 (55.0–74.9) |
|  | NAT-TG3: Failure to undergo curative surgery | 57 | 15.0 (11.0–18.0) | 28.1 (18.5–42.5) | 18.0 (10.2–31.9) |
| **Intention-to-treat population: OS** | No low SMI | 340 | Not reached (not estimable) | 80.9 (76.8–85.2) | 74.4 (69.7–79.3) |
|  | Low SMI | 121 | 35.0 (28.0–48.0) | 66.1 (58.2–75.1) | 49.4 (41.0–59.5) |
| **Surgical population: OS** | No low SMI | 308 | Not reached (not estimable) | 85.3 (81.5–89.4) | 77.8 (73.1–82.8) |
|  | Low SMI | 96 | 42.0 (31.9–not estimable) | 72.8 (64.3–82.3) | 55.8 (46.1–67.6) |
| **Surgical population: DFS** | No low SMI | 308 | Not reached (48.0–not estimable) | 77.6 (73.0–82.4) | 69.9 (64.7–75.4) |
|  | Low SMI | 96 | 35.0 (28.9–not estimable) | 66.5 (57.7–76.7) | 49.3 (39.6–61.3) |

Note: Survival rates and 95% confidence intervals were estimated using the Kaplan-Meier method. In the intention-to-treat population, overall survival was calculated from the initiation of neoadjuvant therapy. In the surgical population, overall and disease-free survival were calculated from the date of surgery. Not reached indicates the median survival was not achieved during follow-up. Not estimable indicates the corresponding confidence interval boundary could not be calculated. Abbreviations: CI, confidence interval; DFS, disease-free survival; NAT-TG, neoadjuvant treatment tolerance grade; OS, overall survival.

**Table S3. Dominant reasons for surgical omission stratified by pretreatment low-SMI status**

| Dominant reason for surgical omission | Total, n = 57 (%) | No low SMI, n = 32 (%) | Low SMI, n = 25 (%) | Included in restricted endpoint |
| --- | --- | --- | --- | --- |
| Severe treatment-related toxicity resulting in treatment discontinuation, prolonged delay, or persistent medical unfitness for surgery | 30 (52.6) | 17 (53.1) | 13 (52.0) | Yes |
| Persistent clinical or functional deterioration without progression sufficient to explain surgical omission | 21 (36.8) | 11 (34.4) | 10 (40.0) | Yes |
| Disease progression as the dominant reason for surgical omission | 6 (10.5) | 4 (12.5) | 2 (8.0) | No |
| Total | 57 (100.0) | 32 (100.0) | 25 (100.0) | — |

Note: Analysis restricted to patients in the intention-to-treat cohort who did not undergo surgical resection. The restricted treatment tolerance/feasibility endpoint captures severe treatment-related toxicity or functional deterioration, excluding surgical omissions primarily attributable to tumor progression.

**Table S4. Sensitivity analysis of baseline factors associated with the restricted neoadjuvant treatment tolerance/feasibility endpoint**

| **Variable** | **Univariable analysis** | | | |  | **Multivariable analysis** | | | |
| --- | --- | --- | --- | --- | --- | --- | --- | --- | --- |
|  | **OR (95% CI)** | ***P* value** | | |  | **OR (95% CI)** | ***P* value** | | |
| Sex |  |  |  |  |  | | |  |  |
| Male | 1.00 (Reference) |  |  |  |  | | |  |  |
| Female | 1.07 (0.66 – 1.72) | 0.785 |  |  |  | | |  |  |
| ECOG |  |  |  |  |  | | |  |  |
| ECOG 0 | 1.00 (Reference) |  |  |  |  | | |  |  |
| ECOG ≥1 | 1.32 (0.88 – 1.96) | 0.175 |  |  |  | | |  |  |
| BMI |  |  |  |  |  | | |  |  |
| <18.5 kg/m^2^ | 1.00 (Reference) |  |  |  | 1.00 (Reference) | | |  |  |
| 18.5–25 kg/m^2^ | 0.52 (0.30 – 0.90) | 0.020 |  |  | 0.57 (0.33 – 1.02) | | | 0.057 |  |
| ≥25 kg/m^2^ | 0.33 (0.15 – 0.72) | 0.005 |  |  | 0.49 (0.22 – 1.10) | | | 0.083 |  |
| Low SMI |  |  |  |  |  | | |  |  |
| No | 1.00 (Reference) |  |  |  | 1.00 (Reference) | | |  |  |
| Yes | 2.87 (1.86 – 4.42) | < 0.001 |  |  | 2.51 (1.59 – 3.95) | | | < 0.001 |  |
| Age (years) |  |  |  |  |  | | |  |  |
| ≤ 60 | 1.00 (Reference) |  |  |  |  | | |  |  |
| > 60 | 1.47 (0.99 – 2.19) | 0.055 |  |  |  | | |  |  |
| Smoking |  |  |  |  |  | | |  |  |
| No | 1.00 (Reference) |  |  |  |  | | |  |  |
| Yes | 1.26 (0.85 – 1.88) | 0.248 |  |  |  | | |  |  |
| Drinking |  |  |  |  |  | | |  |  |
| No | 1.00 (Reference) |  |  |  |  | | |  |  |
| Yes | 0.87 (0.58 – 1.29) | 0.484 |  |  |  | | |  |  |
| Hypertension |  |  |  |  |  | | |  |  |
| No | 1.00 (Reference) |  |  |  |  | | |  |  |
| Yes | 1.11 (0.61 – 2.04) | 0.733 |  |  |  | | |  |  |
| Diabetes |  |  |  |  |  | | |  |  |
| No | 1.00 (Reference) |  |  |  |  | | |  |  |
| Yes | 0.87 (0.42 – 1.81) | 0.705 |  |  |  | | |  |  |
| Tumor location |  |  |  |  |  | | |  |  |
| Upper | 1.00 (Reference) |  |  |  |  | | |  |  |
| Middle | 0.81 (0.47 – 1.42) | 0.463 |  |  |  | | |  |  |
| Lower | 0.78 (0.42 – 1.45) | 0.433 |  |  |  | | |  |  |
| Tumor size |  |  |  |  |  | | |  |  |
| >5 cm | 1.00 (Reference) |  |  |  |  | | |  |  |
| ≤5 cm | 1.14 (0.77 – 1.68) | 0.524 |  |  |  | | |  |  |
| cT stage |  |  |  |  |  | | |  |  |
| T 1–2 | 1.00 (Reference) |  |  |  | 1.00 (Reference) | | |  |  |
| T 3–4 | 1.95 (1.18 – 3.20) | 0.009 |  |  | 1.88 (1.13 – 3.13) | | | 0.016 |  |
| cN stage |  |  |  |  |  | | |  |  |
| N 0 | 1.00 (Reference) |  |  |  |  | | |  |  |
| N + | 1.39 (0.82 – 2.37) | 0.225 |  |  |  | | |  |  |

Note: Patients whose dominant reason for surgical omission was disease progression were excluded. This analysis evaluated the restricted treatment tolerance/feasibility endpoint comprising treatment modification, severe treatment-related toxicity, and persistent clinical or functional deterioration after excluding progression-related surgical omissions.

**Table S5. Baseline characteristics of the surgical population stratified by low-SMI status**

| **Variables** | **Total**  **(n = 404)** | **No low SMI**  **(n = 308)** | **Low SMI**  **(n = 96)** | ***P*** |
| --- | --- | --- | --- | --- |
|  |  |  |  |  |
| Age, Mean ± SD | 61.00 ± 7.26 | 60.87 ± 7.16 | 61.41 ± 7.60 | 0.528 |
| BMI, n (%) |  |  |  | < 0.001 |
| ＜18.5 | 58 (14.36) | 35 (11.36) | 23 (23.96) |  |
| 18.5-25 | 293 (72.52) | 221 (71.75) | 72 (75.00) |  |
| ≥25 | 53 (13.12) | 52 (16.88) | 1 (1.04) |  |
| L3 SMA, Mean ± SD | 151.31 ± 30.92 | 158.59 ± 29.30 | 127.99 ± 23.68 | < 0.001 |
| L3 SMI, Mean ± SD | 54.79 ± 9.79 | 57.75 ± 8.68 | 45.29 ± 6.60 | < 0.001 |
| NAT-TG, n (%) |  |  |  | < 0.001 |
| TG1 | 307 (75.99) | 248 (80.52) | 59 (61.46) |  |
| TG2 | 97 (24.01) | 60 (19.48) | 37 (38.54) |  |
| ECOG, n (%) |  |  |  | 0.966 |
| ECOG 0 | 176 (43.56) | 134 (43.51) | 42 (43.75) |  |
| ECOG ≥1 | 228 (56.44) | 174 (56.49) | 54 (56.25) |  |
| Sex, n (%) |  |  |  | 0.508 |
| Male | 317 (78.47) | 244 (79.22) | 73 (76.04) |  |
| Female | 87 (21.53) | 64 (20.78) | 23 (23.96) |  |
| Smoking, n (%) |  |  |  | 0.869 |
| No | 178 (44.06) | 135 (43.83) | 43 (44.79) |  |
| Yes | 226 (55.94) | 173 (56.17) | 53 (55.21) |  |
| Drinking, n (%) |  |  |  | 0.383 |
| No | 226 (55.94) | 176 (57.14) | 50 (52.08) |  |
| Yes | 178 (44.06) | 132 (42.86) | 46 (47.92) |  |
| Hypertension, n (%) |  |  |  | 0.694 |
| No | 358 (88.61) | 274 (88.96) | 84 (87.50) |  |
| Yes | 46 (11.39) | 34 (11.04) | 12 (12.50) |  |
| Diabetes, n (%) |  |  |  | 0.357 |
| No | 371 (91.83) | 285 (92.53) | 86 (89.58) |  |
| Yes | 33 (8.17) | 23 (7.47) | 10 (10.42) |  |
| cT stage, n (%) |  |  |  | 0.629 |
| 1 | 12 (2.97) | 9 (2.92) | 3 (3.12) |  |
| 2 | 94 (23.27) | 76 (24.68) | 18 (18.75) |  |
| 3 | 206 (50.99) | 156 (50.65) | 50 (52.08) |  |
| 4 | 92 (22.77) | 67 (21.75) | 25 (26.04) |  |
| cN stage, n (%) |  |  |  | 0.826 |
| 0 | 75 (18.56) | 60 (19.48) | 15 (15.62) |  |
| 1 | 106 (26.24) | 79 (25.65) | 27 (28.12) |  |
| 2 | 157 (38.86) | 118 (38.31) | 39 (40.62) |  |
| 3 | 66 (16.34) | 51 (16.56) | 15 (15.62) |  |
| cTNM stage, n (%) |  |  |  | 0.324 |
| II | 74 (18.32) | 60 (19.48) | 14 (14.58) |  |
| III | 193 (47.77) | 149 (48.38) | 44 (45.83) |  |
| IVA | 137 (33.91) | 99 (32.14) | 38 (39.58) |  |
| Immunotherapy, n (%) |  |  |  | 0.314 |
| Nivolumab | 32 (7.92) | 23 (7.47) | 9 (9.38) |  |
| Pembrolizumab | 70 (17.33) | 49 (15.91) | 21 (21.88) |  |
| Camrelizumab | 178 (44.06) | 136 (44.16) | 42 (43.75) |  |
| Tislelizumab | 52 (12.87) | 39 (12.66) | 13 (13.54) |  |
| Sintilimab | 72 (17.82) | 61 (19.81) | 11 (11.46) |  |
| ypT stage, n (%) |  |  |  | 0.806 |
| 0 | 88 (21.78) | 71 (23.05) | 17 (17.71) |  |
| 1 | 52 (12.87) | 39 (12.66) | 13 (13.54) |  |
| 2 | 71 (17.57) | 54 (17.53) | 17 (17.71) |  |
| 3 | 184 (45.54) | 138 (44.81) | 46 (47.92) |  |
| 4 | 9 (2.23) | 6 (1.95) | 3 (3.12) |  |
| ypN stage, n (%) |  |  |  | 0.680 |
| 0 | 214 (52.97) | 168 (54.55) | 46 (47.92) |  |
| 1 | 110 (27.23) | 81 (26.30) | 29 (30.21) |  |
| 2 | 66 (16.34) | 48 (15.58) | 18 (18.75) |  |
| 3 | 14 (3.47) | 11 (3.57) | 3 (3.12) |  |
| ypTNM stage, n (%) |  |  |  | 0.741 |
| I | 140 (34.65) | 109 (35.39) | 31 (32.29) |  |
| II | 68 (16.83) | 54 (17.53) | 14 (14.58) |  |
| III | 179 (44.31) | 133 (43.18) | 46 (47.92) |  |
| IVA | 17 (4.21) | 12 (3.90) | 5 (5.21) |  |
| Differentiation, n (%) |  |  |  | 0.434 |
| G1 | 123 (30.45) | 98 (31.82) | 25 (26.04) |  |
| G2 | 203 (50.25) | 154 (50.00) | 49 (51.04) |  |
| G3 | 78 (19.31) | 56 (18.18) | 22 (22.92) |  |
| TRG, n (%) |  |  |  | 0.053 |
| TRG 0-1 | 169 (41.83) | 137 (44.48) | 32 (33.33) |  |
| TRG 2-3 | 235 (58.17) | 171 (55.52) | 64 (66.67) |  |
| Adjuvant immunotherapy, n (%) |  |  |  | 0.317 |
| No | 165 (40.84) | 130 (42.21) | 35 (36.46) |  |
| Yes | 239 (59.16) | 178 (57.79) | 61 (63.54) |  |

Note: Categorical variables are presented as n (%), and continuous variables as mean ± standard deviation or median (interquartile range), as appropriate. Abbreviations: BMI, body mass index; ECOG, Eastern Cooperative Oncology Group; NAT-TG, neoadjuvant treatment tolerance grade; SMA, skeletal muscle area; SMI, skeletal muscle index; TRG, tumor regression grade; ypTNM, post-neoadjuvant pathological tumor–node–metastasis stage.

**Table S6. Comparison of postoperative complications stratified by pretreatment low-SMI status**

| **Variables** | **Total**  **(n = 404)** | **No low SMI**  **(n = 308)** | **Low SMI**  **(n = 96)** | ***P*** |
| --- | --- | --- | --- | --- |
|  |  |  |  |  |
| Postoperative hospital stay,  Mean ± SD | 12.01 ± 7.58 | 11.67 ± 7.06 | 13.10 ± 9.01 | 0.106 |
| ICU admission, n (%) | 23 (5.69) | 14 (4.55) | 9 (9.38) | 0.075 |
| 30-day readmission, n (%) | 14 (3.47) | 10 (3.25) | 4 (4.17) | 0.912 |
| 30-day mortality, n (%) | 0 (0.0) | 0 (0.0) | 0 (0.0) | — |
| Clavien–Dindo, n (%) |  |  |  | 0.002 |
| None | 188 (46.53) | 156 (50.65) | 32 (33.33) |  |
| I–II | 160 (39.60) | 118 (38.31) | 42 (43.75) |  |
| ≥ III | 56 (13.86) | 34 (11.04) | 22 (22.92) |  |
| Pneumonia, n (%) |  |  |  | 0.006 |
| None | 287 (71.04) | 228 (74.03) | 59 (61.46) |  |
| I–II | 92 (22.77) | 67 (21.75) | 25 (26.04) |  |
| ≥ III | 25 (6.19) | 13 (4.22) | 12 (12.50) |  |
| Anastomotic leak, n (%) |  |  |  | 0.186 |
| None | 356 (88.12) | 276 (89.61) | 80 (83.33) |  |
| I–II | 36 (8.91) | 25 (8.12) | 11 (11.46) |  |
| ≥ III | 12 (2.97) | 7 (2.27) | 5 (5.21) |  |
| Pleural effusion, n (%) |  |  |  | 0.007 |
| None | 239 (59.16) | 193 (62.66) | 46 (47.92) |  |
| I–II | 123 (30.45) | 90 (29.22) | 33 (34.38) |  |
| ≥ III | 42 (10.40) | 25 (8.12) | 17 (17.71) |  |
| Cardiac arrhythmia, n (%) |  |  |  | 0.873 |
| None | 376 (93.07) | 287 (93.18) | 89 (92.71) |  |
| I–II | 28 (6.93) | 21 (6.82) | 7 (7.29) |  |
| Incisional complications, n (%) |  |  |  | 0.068 |
| None | 392 (97.03) | 302 (98.05) | 90 (93.75) |  |
| I–II | 12 (2.97) | 6 (1.95) | 6 (6.25) |  |

Note: Postoperative complications were graded according to the Clavien–Dindo classification. For complications presented in three severity categories, P values reflect the overall comparison of severity distributions between groups.

**Table S7. Univariable and multivariable logistic regression analyses of predictors for severe postoperative complications**

|  | **Univariable analysis** | | | **Multivariable analysis** | | |
| --- | --- | --- | --- | --- | --- | --- |
| **Characteristic** | **OR** | **95% CI** | ***P* value** | **OR** | **95% CI** | ***P* value** |
| Low SMI |  |  |  |  |  |  |
| No | 1.00 (Reference) | — |  | 1.00 (Reference) | — |  |
| Yes | 2.40 | 1.31, 4.32 | 0.004 | 2.92 | 1.51, 5.63 | 0.001 |
| Age |  |  |  |  |  |  |
| ≤ 60 | 1.00 (Reference) | — |  | 1.00 (Reference) | — |  |
| ＞60 | 1.43 | 0.81, 2.57 | 0.216 | 1.57 | 0.87, 2.89 | 0.141 |
| Sex |  |  |  |  |  |  |
| Male | 1.00 (Reference) | — |  | 1.00 (Reference) | — |  |
| Female | 0.99 | 0.48, 1.92 | 0.983 | 0.90 | 0.42, 1.80 | 0.774 |
| BMI |  |  |  |  |  |  |
| ＜18.5 kg/m^2^ | 1.00 (Reference) | — |  | 1.00 (Reference) | — |  |
| 18.5–25 kg/m^2^ | 0.79 | 0.37, 1.83 | 0.552 | 1.01 | 0.45, 2.45 | 0.990 |
| ≥25 kg/m^2^ | 1.27 | 0.47, 3.47 | 0.640 | 2.63 | 0.86, 8.29 | 0.092 |
| Serum Albumin (g/L) | 1.0 | 0.92, 1.07 | 0.888 | 1.01 | 0.93, 1.10 | 0.833 |
| Operative Approach |  |  |  |  |  |  |
| Thoracoscopic minimally invasive | 1.00 (Reference) | — |  | 1.00 (Reference) | — |  |
| Robotic minimally invasive | 0.92 | 0.44, 1.82 | 0.829 | 0.87 | 0.39, 1.81 | 0.729 |
| Open | 1.23 | 0.06, 7.85 | 0.853 | 1.29 | 0.06, 9.51 | 0.827 |
| Lymphadenectomy Extent |  |  |  |  |  |  |
| Two-field | 1.00 (Reference) | — |  | 1.00 (Reference) | — |  |
| Three-field | 1.06 | 0.51, 2.06 | 0.860 | 1.13 | 0.53, 2.28 | 0.736 |
| Tumor location |  |  |  |  |  |  |
| Upper | 1.00 (Reference) | — |  | 1.00 (Reference) | — |  |
| Middle | 0.87 | 0.39, 2.15 | 0.753 | 0.72 | 0.31, 1.83 | 0.465 |
| Lower | 1.15 | 0.48, 2.97 | 0.763 | 1.02 | 0.41, 2.76 | 0.962 |

Note: The multivariable logistic regression evaluated predictors of severe postoperative complications, defined as Clavien-Dindo grade ≥III. The multivariable model adjusted for age, sex, BMI, serum albumin, operative approach, lymphadenectomy extent, tumor location, and participating center, with center entered as a fixed effect.

**Table S8. Univariable and multivariable Cox proportional hazards analyses for overall survival in the intention-to-treat population**

|  | **Univariable analysis** | | | **Model 1** | | | **Model 2 (primary)** | | | **Model 3 (sensitivity)** | | |
| --- | --- | --- | --- | --- | --- | --- | --- | --- | --- | --- | --- | --- |
| **Variable** | **HR** | **95% CI** | ***P* value** | **HR** | **95% CI** | ***P* value** | **HR** | **95% CI** | ***P* value** | **HR** | **95% CI** | ***P* value** |
| Low SMI |  |  |  |  |  |  |  |  |  |  |  |  |
| No | 1.00 (Reference) | — |  | 1.00 (Reference) | — |  | 1.00 (Reference) | — |  | 1.00 (Reference) | — |  |
| Yes | 2.38 | 1.74, 3.25 | <0.001 | 2.17 | 1.56, 3.00 | <0.001 | 2.36 | 1.67, 3.33 | <0.001 | 2.38 | 1.68, 3.38 | <0.001 |
| Age, years |  |  |  |  |  |  |  |  |  |  |  |  |
| ≤60 | 1.00 (Reference) | — |  |  |  |  | 1.00 (Reference) | — |  | 1.00 (Reference) | — |  |
| >60 | 1.18 | 0.87, 1.60 | 0.300 |  |  |  | 1.20 | 0.87, 1.66 | 0.264 | 1.16 | 0.84, 1.61 | 0.365 |
| Sex |  |  |  |  |  |  |  |  |  |  |  |  |
| Male | 1.00 (Reference) | — |  |  |  |  | 1.00 (Reference) | — |  | 1.00 (Reference) | — |  |
| Female | 0.74 | 0.49, 1.10 | 0.133 |  |  |  | 0.73 | 0.48, 1.09 | 0.126 | 0.71 | 0.47, 1.08 | 0.111 |
| BMI, kg/m² |  |  |  |  |  |  |  |  |  |  |  |  |
| 18.5–<25 | 1.00 (Reference) | — |  |  |  |  | 1.00 (Reference) | — |  | 1.00 (Reference) | — |  |
| <18.5 | 1.06 | 0.68, 1.64 | 0.801 |  |  |  | 0.84 | 0.53, 1.34 | 0.459 | 0.81 | 0.50, 1.30 | 0.376 |
| ≥25 | 1.04 | 0.66, 1.64 | 0.864 |  |  |  | 1.47 | 0.90, 2.41 | 0.121 | 1.51 | 0.92, 2.47 | 0.105 |
| ECOG performance status |  |  |  |  |  |  |  |  |  |  |  |  |
| 0 | 1.00 (Reference) | — |  |  |  |  | 1.00 (Reference) | — |  | 1.00 (Reference) | — |  |
| ≥1 | 1.09 | 0.80, 1.48 | 0.584 |  |  |  | 0.96 | 0.69, 1.34 | 0.825 | 0.96 | 0.69, 1.34 | 0.828 |
| Clinical TNM stage |  |  |  |  |  |  |  |  |  |  |  |  |
| II | 1.00 (Reference) | — |  |  |  |  | 1.00 (Reference) | — |  | 1.00 (Reference) | — |  |
| III | 0.87 | 0.57, 1.34 | 0.528 |  |  |  | 0.85 | 0.55, 1.32 | 0.481 | 0.86 | 0.55, 1.33 | 0.490 |
| IVA | 1.20 | 0.78, 1.85 | 0.404 |  |  |  | 1.07 | 0.68, 1.68 | 0.762 | 1.12 | 0.71, 1.77 | 0.616 |
| Albumin, per 1-g/L increase | 0.92 | 0.89, 0.95 | <0.001 | 0.93 | 0.90, 0.95 | <0.001 | 0.93 | 0.90, 0.96 | <0.001 | 0.93 | 0.90, 0.96 | <0.001 |
| SII, per twofold increase | 1.25 | 1.03, 1.53 | 0.024 | 1.12 | 0.92, 1.35 | 0.259 | 1.14 | 0.94, 1.38 | 0.189 | 1.10 | 0.90, 1.34 | 0.372 |
| Neoadjuvant treatment cycles |  |  |  |  |  |  |  |  |  |  |  |  |
| 2–4 cycles | 1.00 (Reference) | — |  |  |  |  |  |  |  | 1.00 (Reference) | — |  |
| 1 cycle | 1.68 | 1.08, 2.64 | 0.023 |  |  |  |  |  |  | 1.57 | 0.98, 2.53 | 0.061 |
| PD-1 inhibitor |  |  |  |  |  |  |  |  |  |  |  |  |
| Camrelizumab | 1.00 (Reference) | — |  |  |  |  |  |  |  | 1.00 (Reference) | — |  |
| Nivolumab | 1.12 | 0.66, 1.89 | 0.683 |  |  |  |  |  |  | 1.45 | 0.82, 2.55 | 0.197 |
| Pembrolizumab | 0.97 | 0.62, 1.53 | 0.911 |  |  |  |  |  |  | 1.11 | 0.70, 1.76 | 0.663 |
| Tislelizumab | 0.98 | 0.61, 1.57 | 0.936 |  |  |  |  |  |  | 0.99 | 0.59, 1.65 | 0.961 |
| Sintilimab | 0.85 | 0.55, 1.33 | 0.479 |  |  |  |  |  |  | 1.16 | 0.73, 1.84 | 0.536 |

Note: CI, confidence interval; HR, hazard ratio. Model 1 used a data-driven approach in which low SMI was included a priori and other baseline pretreatment variables with a univariable global P value <0.10 were selected; the resulting model included serum albumin and log₂-transformed systemic immune-inflammation index. Model 2, the primary clinically specified model, was adjusted for age, sex, body mass index, ECOG performance status, clinical TNM stage, serum albumin, and log₂-transformed systemic immune-inflammation index. Model 3, the sensitivity model, additionally included the actual number of nICT cycles and PD-1 inhibitor type. All multivariable models were stratified by participating center. The intention-to-treat cohort comprised 461 patients and 165 deaths. Models 1, 2, and 3 included 3, 10, and 15 regression parameters, corresponding to 55.0, 16.5, and 11.0 events per parameter, respectively. Global proportional hazards tests based on Schoenfeld residuals yielded P values of 0.950, 0.430, and 0.620, respectively, indicating no evidence of violation of the proportional hazards assumption.

**Table S9. Univariable Cox proportional hazards analysis of treatment-related characteristics for overall survival**

| **Characteristic** | **Univariable analysis** | | |
| --- | --- | --- | --- |
|  | **HR** | **95% CI** | ***P* value** |
| Neoadjuvant Cycle Protocol |  |  |  |
| 3 cycles | 1.00 (Reference) | — |  |
| 2 cycles | 1.17 | 0.77 – 1.77 | 0.473 |
| Year of Treatment |  |  |  |
| 2020–2021 | 1.00 (Reference) | — |  |
| 2022–2023 | 0.99 | 0.73 – 1.35 | 0.956 |
| Taxane Backbone |  |  |  |
| Paclitaxel | 1.00 (Reference) | — |  |
| Nab-paclitaxel | 1.02 | 0.72 – 1.42 | 0.931 |
| Platinum Backbone |  |  |  |
| Cisplatin | 1.00 (Reference) | — |  |
| Carboplatin | 1.21 | 0.84 – 1.74 | 0.296 |
| Nedaplatin | 0.84 | 0.56 – 1.25 | 0.390 |
| Actual Neoadjuvant cycles |  |  |  |
| ≥ 2 cycles | 1.00 (Reference) | — |  |
| 1 cycle | 1.68 | 1.08, 2.64 | 0.023 |
| PD-1 inhibitor |  |  |  |
| Camrelizumab | 1.00 (Reference) | — |  |
| Nivolumab | 1.12 | 0.66, 1.89 | 0.683 |
| Pembrolizumab | 0.97 | 0.62, 1.53 | 0.911 |
| Tislelizumab | 0.98 | 0.61, 1.57 | 0.936 |
| Sintilimab | 0.85 | 0.55, 1.33 | 0.479 |

Note: Univariable Cox regression was used to evaluate the associations of treatment year, planned-cycle protocol, PD-1 inhibitor type, chemotherapy backbone, and actual cycles received with overall survival in the intention-to-treat population.

**Table S10. Sensitivity analyses using study-derived and published definitions of low L3 skeletal muscle index**

| **Definition** | **Low SMI, n (%)** | **Male, n/N (%)** | **Female, n/N (%)** | **Unfavorable NAT-TG, adjusted OR** | **ITT OS, Model 2 adjusted HR** | **ITT OS, Model 3 adjusted HR** | **Surgical OS, adjusted HR** |
| --- | --- | --- | --- | --- | --- | --- | --- |
| Study-derived cutoff | 121/461 (26.2) | 92/363 (25.3) | 29/98 (29.6) | 2.48 (1.62–3.82); < 0.001 | 2.36 (1.67–3.33); < 0.001 | 2.38 (1.68–3.38); < 0.001 | 1.91 (1.31–2.80); < 0.001 |
| Prado | 137/461 (29.7) | 106/363 (29.2) | 31/98 (31.6) | 2.44 (1.57–3.78); < 0.001 | 1.98 (1.41–2.78); < 0.001 | 1.94 (1.37–2.73); < 0.001 | 1.65 (1.13 – 2.41); 0.010 |
| Fearon | 185/461 (40.1) | 149/363 (41.0) | 36/98 (36.7) | 1.95 (1.28–2.96); 0.002 | 1.50 (1.09–2.08); 0.013 | 1.48 (1.07–2.05); 0.019 | 1.37 (0.95–1.99); 0.091 |

Note: The study-derived cutoff was determined using X-tile analysis, whereas the Prado and Fearon cutoffs were based on previously published definitions.

**Table S11. Comparison of baseline characteristics between patients receiving and not receiving adjuvant immunotherapy, stratified by low-SMI status**

| **Variables** | **Low SMI**  **(n = 96)** | **Observation**  **(n = 35)** | **Adjuvant immunotherapy**  **(n = 61)** | ***P*** | **No low SMI**  **(n = 308)** | **Observation**  **(n = 130)** | **Adjuvant immunotherapy**  **(n = 178)** | ***P*** |
| --- | --- | --- | --- | --- | --- | --- | --- | --- |
| Age, Mean ± SD | 61.41 ± 7.60 | 62.11 ± 8.32 | 61.00 ± 7.19 | 0.492 | 60.87 ± 7.16 | 61.89 ± 7.22 | 60.12 ± 7.04 | 0.032 |
| BMI, n (%) |  |  |  | 0.134 |  |  |  | 0.713 |
| ＜18.5 | 23 (23.96) | 12 (34.29) | 11 (18.03) |  | 35 (11.36) | 17 (13.08) | 18 (10.11) |  |
| 18.5–25 | 72 (75.00) | 23 (65.71) | 49 (80.33) |  | 221 (71.75) | 91 (70.00) | 130 (73.03) |  |
| ≥25 | 1 (1.04) | 0 (0.00) | 1 (1.64) |  | 52 (16.88) | 22 (16.92) | 30 (16.85) |  |
| Sex, n (%) |  |  |  | 0.760 |  |  |  | 0.997 |
| Male | 73 (76.04) | 26 (74.29) | 47 (77.05) |  | 244 (79.22) | 103 (79.23) | 141 (79.21) |  |
| Female | 23 (23.96) | 9 (25.71) | 14 (22.95) |  | 64 (20.78) | 27 (20.77) | 37 (20.79) |  |
| ECOG, n (%) |  |  |  | 0.323 |  |  |  | 0.079 |
| ECOG 0 | 42 (43.75) | 13 (37.14) | 29 (47.54) |  | 134 (43.51) | 49 (37.69) | 85 (47.75) |  |
| ECOG ≥1 | 54 (56.25) | 22 (62.86) | 32 (52.46) |  | 174 (56.49) | 81 (62.31) | 93 (52.25) |  |
| Smoking, n (%) |  |  |  | 0.322 |  |  |  | 0.247 |
| No | 43 (44.79) | 18 (51.43) | 25 (40.98) |  | 135 (43.83) | 52 (40.00) | 83 (46.63) |  |
| Yes | 53 (55.21) | 17 (48.57) | 36 (59.02) |  | 173 (56.17) | 78 (60.00) | 95 (53.37) |  |
| Drinking, n (%) |  |  |  | 0.191 |  |  |  | 0.338 |
| No | 41 (42.71) | 18 (51.43) | 23 (37.70) |  | 152 (49.35) | 60 (46.15) | 92 (51.69) |  |
| Yes | 55 (57.29) | 17 (48.57) | 38 (62.30) |  | 156 (50.65) | 70 (53.85) | 86 (48.31) |  |
| Hypertension, n (%) |  |  |  | 1.000 |  |  |  | 0.209 |
| No | 84 (87.50) | 31 (88.57) | 53 (86.89) |  | 282 (91.56) | 116 (89.23) | 166 (93.26) |  |
| Yes | 12 (12.50) | 4 (11.43) | 8 (13.11) |  | 26 (8.44) | 14 (10.77) | 12 (6.74) |  |
| Diabetes, n (%) |  |  |  | 1.000 |  |  |  | 0.385 |
| No | 92 (95.83) | 34 (97.14) | 58 (95.08) |  | 295 (95.78) | 123 (94.62) | 172 (96.63) |  |
| Yes | 4 (4.17) | 1 (2.86) | 3 (4.92) |  | 13 (4.22) | 7 (5.38) | 6 (3.37) |  |
| cT stage, n (%) |  |  |  | 0.913 |  |  |  | 0.358 |
| 1 | 3 (3.12) | 1 (2.86) | 2 (3.28) |  | 9 (2.92) | 5 (3.85) | 4 (2.25) |  |
| 2 | 18 (18.75) | 6 (17.14) | 12 (19.67) |  | 76 (24.68) | 37 (28.46) | 39 (21.91) |  |
| 3 | 50 (52.08) | 20 (57.14) | 30 (49.18) |  | 156 (50.65) | 59 (45.38) | 97 (54.49) |  |
| 4 | 25 (26.04) | 8 (22.86) | 17 (27.87) |  | 67 (21.75) | 29 (22.31) | 38 (21.35) |  |
| cN stage, n (%) |  |  |  | 0.181 |  |  |  | 0.077 |
| 0 | 15 (15.62) | 5 (14.29) | 10 (16.39) |  | 60 (19.48) | 32 (24.62) | 28 (15.73) |  |
| 1 | 27 (28.12) | 14 (40.00) | 13 (21.31) |  | 79 (25.65) | 25 (19.23) | 54 (30.34) |  |
| 2 | 39 (40.62) | 10 (28.57) | 29 (47.54) |  | 118 (38.31) | 50 (38.46) | 68 (38.20) |  |
| 3 | 15 (15.62) | 6 (17.14) | 9 (14.75) |  | 51 (16.56) | 23 (17.69) | 28 (15.73) |  |
| cTNM stage, n (%) |  |  |  | 0.997 |  |  |  | 0.090 |
| II | 14 (14.58) | 5 (14.29) | 9 (14.75) |  | 60 (19.48) | 32 (24.62) | 28 (15.73) |  |
| III | 44 (45.83) | 16 (45.71) | 28 (45.90) |  | 149 (48.38) | 55 (42.31) | 94 (52.81) |  |
| IVA | 38 (39.58) | 14 (40.00) | 24 (39.34) |  | 99 (32.14) | 43 (33.08) | 56 (31.46) |  |
| NAT-TG, n (%) |  |  |  | 0.824 |  |  |  | 0.844 |
| TG1 | 59 (61.46) | 21 (60.00) | 38 (62.30) |  | 248 (80.52) | 104 (80.00) | 144 (80.90) |  |
| TG2 | 37 (38.54) | 14 (40.00) | 23 (37.70) |  | 60 (19.48) | 26 (20.00) | 34 (19.10) |  |
| Cycle, n (%) |  |  |  | 0.734 |  |  |  | 0.693 |
| 2–4 cycle | 85 (88.54) | 32 (91.43) | 53 (86.89) |  | 287 (93.18) | 122 (93.85) | 165 (92.70) |  |
| ≤1 cycle | 11 (11.46) | 3 (8.57) | 8 (13.11) |  | 21 (6.82) | 8 (6.15) | 13 (7.30) |  |
| TRG, n (%) |  |  |  | 0.453 |  |  |  | 0.005 |
| TRG 0-1 | 32 (33.33) | 10 (28.57) | 22 (36.07) |  | 137 (44.48) | 70 (53.85) | 67 (37.64) |  |
| TRG 2-3 | 64 (66.67) | 25 (71.43) | 39 (63.93) |  | 171 (55.52) | 60 (46.15) | 111 (62.36) |  |
| ypT stage, n (%) |  |  |  | 0.949 |  |  |  | 0.002 |
| 0 | 17 (17.71) | 6 (17.14) | 11 (18.03) |  | 71 (23.05) | 41 (31.54) | 30 (16.85) |  |
| 1 | 13 (13.54) | 6 (17.14) | 7 (11.48) |  | 39 (12.66) | 22 (16.92) | 17 (9.55) |  |
| 2 | 17 (17.71) | 6 (17.14) | 11 (18.03) |  | 54 (17.53) | 18 (13.85) | 36 (20.22) |  |
| 3 | 46 (47.92) | 16 (45.71) | 30 (49.18) |  | 138 (44.81) | 48 (36.92) | 90 (50.56) |  |
| 4 | 3 (3.12) | 1 (2.86) | 2 (3.28) |  | 6 (1.95) | 1 (0.77) | 5 (2.81) |  |
| ypN stage, n (%) |  |  |  | 0.749 |  |  |  | 0.004 |
| 0 | 46 (47.92) | 18 (51.43) | 28 (45.90) |  | 168 (54.55) | 84 (64.62) | 84 (47.19) |  |
| 1 | 29 (30.21) | 11 (31.43) | 18 (29.51) |  | 81 (26.30) | 29 (22.31) | 52 (29.21) |  |
| 2 | 18 (18.75) | 6 (17.14) | 12 (19.67) |  | 48 (15.58) | 11 (8.46) | 37 (20.79) |  |
| 3 | 3 (3.12) | 0 (0.00) | 3 (4.92) |  | 11 (3.57) | 6 (4.62) | 5 (2.81) |  |
| ypTNM stage, n (%) |  |  |  | 0.834 |  |  |  | < 0.001 |
| I | 31 (32.29) | 13 (37.14) | 18 (29.51) |  | 109 (35.39) | 60 (46.15) | 49 (27.53) |  |
| II | 14 (14.58) | 5 (14.29) | 9 (14.75) |  | 54 (17.53) | 24 (18.46) | 30 (16.85) |  |
| III | 46 (47.92) | 16 (45.71) | 30 (49.18) |  | 133 (43.18) | 39 (30.00) | 94 (52.81) |  |
| IVA | 5 (5.21) | 1 (2.86) | 4 (6.56) |  | 12 (3.90) | 7 (5.38) | 5 (2.81) |  |
| Differentiation, n (%) |  |  |  | 0.492 |  |  |  | 0.363 |
| G1 | 25 (26.04) | 11 (31.43) | 14 (22.95) |  | 98 (31.82) | 47 (36.15) | 51 (28.65) |  |
| G2 | 49 (51.04) | 18 (51.43) | 31 (50.82) |  | 154 (50.00) | 60 (46.15) | 94 (52.81) |  |
| G3 | 22 (22.92) | 6 (17.14) | 16 (26.23) |  | 56 (18.18) | 23 (17.69) | 33 (18.54) |  |

**Table S12. Univariable and multivariable Cox proportional hazards analyses for overall survival and disease-free survival within the low-SMI subgroup**

| **Variables** | **OS** | | | | |  | **DFS** | | | | |
| --- | --- | --- | --- | --- | --- | --- | --- | --- | --- | --- | --- |
|  |  | **Univariable analysis** |  | **Multivariable analysis** |  |  |  | **Univariable analysis** |  | **Multivariable analysis** |  |
|  |  | **HR (95% CI)** | ***P*** | **HR (95% CI)** | ***P*** |  |  | **HR (95% CI)** | ***P*** | **HR (95% CI)** | ***P*** |
| Age |  |  |  |  |  |  |  |  |  |  |  |
| ≤ 60 |  | 1.00 (Reference) |  | 1.00 (Reference) |  |  |  | 1.00 (Reference) |  | 1.00 (Reference) |  |
| > 60 |  | 1.31 (0.72 – 2.40) | 0.377 | 1.31 (0.69 – 2.50) | 0.405 |  |  | 1.51 (0.84 – 2.69) | 0.166 | 1.70 (0.89 – 3.24) | 0.109 |
| Sex |  |  |  |  |  |  |  |  |  |  |  |
| Male |  | 1.00 (Reference) |  | 1.00 (Reference) |  |  |  | 1.00 (Reference) |  | 1.00 (Reference) |  |
| Female |  | 1.11 (0.56 – 2.20) | 0.774 | 1.04 (0.52 – 2.09) | 0.913 |  |  | 1.05 (0.55 – 2.03) | 0.875 | 0.87 (0.45 – 1.72) | 0.697 |
| Adjuvant immunotherapy |  |  |  |  |  |  |  |  |  |  |  |
| Observation |  | 1.00 (Reference) |  | 1.00 (Reference) |  |  |  | 1.00 (Reference) |  | 1.00 (Reference) |  |
| Adjuvant |  | 0.53 (0.29 – 0.96) | 0.037 | 0.47 (0.25 – 0.88) | 0.019 |  |  | 0.53 (0.30 – 0.94) | 0.030 | 0.47 (0.26 – 0.87) | 0.016 |
| ypTNM stage |  |  |  |  |  |  |  |  |  |  |  |
| I |  | 1.00 (Reference) |  | 1.00 (Reference) |  |  |  | 1.00 (Reference) |  | 1.00 (Reference) |  |
| II |  | 0.91 (0.28 – 2.96) | 0.876 | 1.25 (0.37 – 4.28) | 0.722 |  |  | 0.74 (0.24 – 2.31) | 0.608 | 1.06 (0.33 – 3.43) | 0.924 |
| III |  | 2.23 (1.05 – 4.74) | 0.038 | 2.60 (1.20 – 5.62) | 0.015 |  |  | 1.92 (0.98 – 3.79) | 0.058 | 2.46 (1.22 – 4.94) | 0.011 |
| IVA |  | 5.30 (1.61 – 17.39) | 0.006 | 9.57 (2.64 – 34.75) | < 0.001 |  |  | 3.87 (1.24 – 12.11) | 0.020 | 7.86 (2.28 – 27.11) | 0.001 |
| Clavien–Dindo |  |  |  |  |  |  |  |  |  |  |  |
| None |  | 1.00 (Reference) |  | 1.00 (Reference) |  |  |  | 1.00 (Reference) |  | 1.00 (Reference) |  |
| I–II |  | 0.82 (0.41 – 1.62) | 0.562 | 0.75 (0.37 – 1.54) | 0.436 |  |  | 0.80 (0.41 – 1.56) | 0.519 | 0.65 (0.32 – 1.32) | 0.231 |
| ≥ III |  | 1.13 (0.52 – 2.44) | 0.756 | 1.16 (0.51 – 2.61) | 0.722 |  |  | 1.31 (0.63 – 2.73) | 0.464 | 1.07 (0.48 – 2.37) | 0.876 |

Note: Analysis was restricted to patients with baseline low SMI. The multivariable models evaluated the association of postoperative adjuvant immunotherapy with survival after adjustment for age, sex, ypTNM stage, and postoperative Clavien–Dindo grade.

**Figure S1. Correlation and discordance between body mass index and skeletal muscle index.** Note: Scatter plots showing the relationship between BMI and L3-SMI stratified by sex. Horizontal dashed lines represent the sex-specific low-SMI cutoff values. BMI, body mass index; SMI, skeletal muscle index.

**Figure S2. Comparative Profile of Adverse Events During Neoadjuvant Immunochemotherapy Between Groups.**

**Figure S3. Bootstrap calibration curves for 3-year overall survival in the intention-to-treat population.** Calibration curves based on 1,000 bootstrap resamples are shown for (A) Model 2, the primary clinically specified model, and (B) Model 3, the extended sensitivity model. The dashed diagonal line represents the ideal reference for perfect calibration. Crosses indicate Kaplan–Meier estimates of observed 3-year OS within predicted-risk strata containing approximately 80 patients each, and vertical bars indicate 95% confidence intervals. Rug marks show the distribution of predicted survival probabilities.

**Figure S4. Subgroup analysis of the association between adjuvant immunotherapy and survival in the surgical population.**

**Figure S5. Exploratory association of adjuvant immunotherapy with survival in patients with low SMI.** (A) Kaplan–Meier curves for OS comparing postoperative adjuvant immunotherapy with observation in the low-SMI subgroup (n = 96). Adjuvant immunotherapy was associated with higher OS (P = 0.033), with 3-year OS rates of 63.8% versus 43.1%, respectively. Among 44 deaths, 23 occurred in the adjuvant immunotherapy group and 21 in the observation group. (B) Kaplan–Meier curves for DFS. Adjuvant immunotherapy was associated with higher DFS (P = 0.027), with 3-year DFS rates of 59.0% versus 34.5%. Among 48 recurrence or death events, 25 occurred in the adjuvant immunotherapy group and 23 in the observation group. (C) Six-month landmark analysis of OS among patients with low SMI who were alive and recurrence-free at 6 months after surgery. The estimate favored adjuvant immunotherapy, although the confidence interval crossed the null (HR, 0.58; 95% CI, 0.31–1.09; P = 0.089). (D) Six-month landmark analysis of DFS among patients alive and recurrence-free at 6 months. The estimate was directionally similar but did not reach statistical significance (HR, 0.56; 95% CI, 0.31–1.02; P = 0.052). Treatment exposure in the landmark analysis was defined using the 3-month postoperative window and was therefore fully ascertained before the 6-month landmark.
